# Carrier Screening and Potential-Difference Response of a Piezomagnetic–Piezoelectric Semiconductor PN-Junction Sandwich Fiber Under a Local Magnetic Field

**DOI:** 10.3390/s26216983

**Published:** 2026-11-04

**Authors:** Chengcheng Liu, Wei Zhou, Yong Ma, Yifan Hu

**Affiliations:** Department of Mechanical and Automotive Engineering, Science and Technology College of Hubei University of Arts and Science, Xiangyang 441025, China

**Keywords:** piezomagnetic–piezoelectric composite, piezoelectric semiconductor, PN junction, local magnetic field, carrier screening, potential-difference response, magnetoelectric sensing

## Abstract

**Highlights:**

**What are the main findings?**

- For the stated reference case, fixed field-edge and junction readouts give about 200 and 50 times the end-to-end potential-difference response, respectively.
- Under equal screening, fixed interior pairs have maximal response when a step-field edge reaches them; layer thickness changes the source and screening.

**What are the implications of the main findings?**

- Local potential variation can exceed the end-to-end signal; readout position and field width must be considered together.
- Under equal screening, a weak PN junction changes the reference field but not the magnetic potential-difference response.

**Abstract:**

Carrier screening and observation geometry determine how a local magnetoelectric response contributes to a potential-difference readout. This study develops a finite-length one-dimensional analytical model for a weak PN-junction piezoelectric-semiconductor fiber between magnetostrictive layers subjected to a localized magnetic-field increment about a prescribed bias. Cross-sectional force balance, two-carrier drift–diffusion, blocking end conditions, and interface continuity yield a four-region solution that separates the junction reference from the magnetic increment. In the representative weak-junction case, the end-to-end difference is about 1% of the peak local magnetically induced potential, whereas observation pairs at the field-region edges and near the junction give differences about 200 and 50 times larger than the end-to-end value. For equal screening and a step field, the response of a fixed interior pair peaks when the field-region edges reach the observation points. Uniformly doped and symmetric weak-junction controls have identical magnetic increments under equal screening despite their different reference fields. Layer thickness modifies both magnetic conversion and carrier screening. The resulting continuum-model relations identify how excitation width, screening length, and observation position jointly govern internal magnetic-field readout.

## 1. Introduction

In piezoelectric semiconductors, mechanical deformation generates polarization, and the associated electric field redistributes mobile carriers. This coupling underlies piezotronic control of charge transport and junction behavior. Experiments on ZnO nanowire arrays established their potential for mechanical-to-electrical conversion [1], while the concept of nanopiezotronics framed strain-induced potential as a means of controlling carrier transport [2]. Subsequent piezopotential-gated devices and theoretical studies clarified the coupled roles of strain, polarization, and transport [3,4,5]. Research on nanowire interfaces, two-dimensional piezoelectrics, and GaN-based devices has broadened the available material and device platforms [6,7,8]. In structures containing interfaces and mobile charge, however, the piezoelectric coefficient alone does not determine the electrical response [9]. For magnetic sensing, the relevant quantity is the portion of the induced potential difference that remains between specified readout locations after carrier screening.

Macroscopic theory provides a framework that couples mechanical equilibrium, Gauss’s law, and drift–diffusion transport [10]. Analytical studies of axially loaded fibers have traced polarization-induced carrier redistribution [11], while nonlinear current models reveal feedback between the carrier profile and the internal electric field [12]. Analyses of beams, vibrating nanowires, and composite sections show that the deformation mode and interlayer compatibility affect the magnitude and spatial distribution of the polarization source [13,14,15,16]. A PN junction adds a built-in field associated with the different reference carrier populations on its two sides. One-dimensional junction analyses [17], studies of mechanically loaded junctions [18], and bending analyses of composite junction fibers [19] have examined this field and its electromechanical modulation. Nonlinear solutions further delineate why weak-perturbation expressions should not be extended directly to strongly depleted junctions or biased operation [20]. The magnetic increment must therefore be evaluated separately from the zero-increment junction baseline.

When excitation is confined to part of a fiber, the distance between the source boundary and the observation point becomes an additional governing length scale. Local stress can produce potential barriers, wells, and carrier accumulation in piezoelectric semiconductor fibers [21], and can alter their biased transport [22]; multiple loading zones can generate interacting potential features [23]. Thermal loading provides a related example. A uniform temperature change modifies the carrier distribution through thermoelastic and pyroelectric coupling [24], whereas a local temperature change produces an electrical response concentrated near the thermal boundaries [25]. Studies of temperature effects and temperature gradients in PN junctions [26,27,28] show that junction behavior depends on both the applied thermal source and the reference carrier distribution. Work on thermopiezoelectric plates and films [29,30], composite fibers [31], gradient-temperature structures [32], nonlinear thermoelectric coupling [33], and locally excited films and plates [34,35] has extended this picture to other geometries. Although the magnetic source considered here has a different physical origin, its electrical effect is likewise shaped by spatial transfer and carrier screening. A pronounced potential variation near the field-region edge need not persist at a remote junction or fiber end.

A magnetostrictive layer provides an indirect route for magnetic control of a piezoelectric semiconductor. In the strict symmetry sense, intrinsic piezomagnetism is a linear magneto-mechanical effect; the coefficient used here for CoFe_2_O_4_ has a different, operational meaning. It is the tangent coefficient of its magnetostrictive response at a prescribed magnetic bias, used only for small field increments about that state. Composite-fiber and rod studies describe how such magnetic strain is transferred through the interface, produces piezoelectric polarization, and redistributes carriers [36,37]. A composite PN-junction fiber has been studied under a magnetic field [38], while a uniformly doped fiber with a spatially restricted field exhibits electrical features near the field-region edges [39]. The uniform-field study found little change in the homojunction but a stronger response in a heterojunction [38]. The present homojunction model instead retains the spatial gradients of the imposed magnetic increment, which generate electric-polarization sources at the edges of the field-excited region. This distinction concerns the excitation geometry, not an intrinsic enhancement of magnetic gain by the PN interface. Studies of time-dependent fields, asymmetric structures, and composite shells extend the available geometries [40,41,42]. Analyses of concurrent local magnetic and thermal fields [43] and a locally heated PN junction [44] further motivate distinguishing excitation edges from the doping interface. The unresolved issue here is how much of the induced potential difference survives between fixed readout points, and whether the weak PN junction changes that increment or chiefly the zero-increment reference field. Unlike the spatially uniform magnetic junction treatment [38], the present problem retains two finite-source edges and compares fixed readout pairs. Unlike the locally magnetized uniformly doped fiber [39], it separates the zero-increment PN reference from the magnetic increment. The local magnetothermal composite study [43] has no PN interface, while the locally heated junction [44] has a different source mechanism. Equal-screening results below apply only when the two reference carrier totals match; the closed-form weak-junction perturbation does not establish behavior for strongly depleted junctions.

We therefore analyze a finite-length, symmetric sandwich fiber under a local transverse magnetic-field increment about a prescribed bias. The model distinguishes the PN doping interface at the fiber center from the two edges of the field-excited region. Carrier-blocking and zero-end-displacement conditions are imposed independently. The four-region solution is evaluated for three fixed readout pairs; uniformly doped and weakly asymmetric controls separate junction-reference effects from magnetic response. We also examine the roles of layer thickness and field-region width and compare the analytical fields with independent numerical and nonlinear drift–diffusion calculations. The intended application is quasi-static magnetic-field sensing, with the internal potential difference between fixed observation points serving as the model readout. The analysis relates this response to carrier screening, excitation width, and layer geometry, thereby informing the choice of observation positions. Electrode contacts and the external readout circuit are outside the present formulation.

## 2. Physical Model and Basic Relations

### 2.1. Sandwich Configuration and Local Magnetic Field

**Figure 1:**
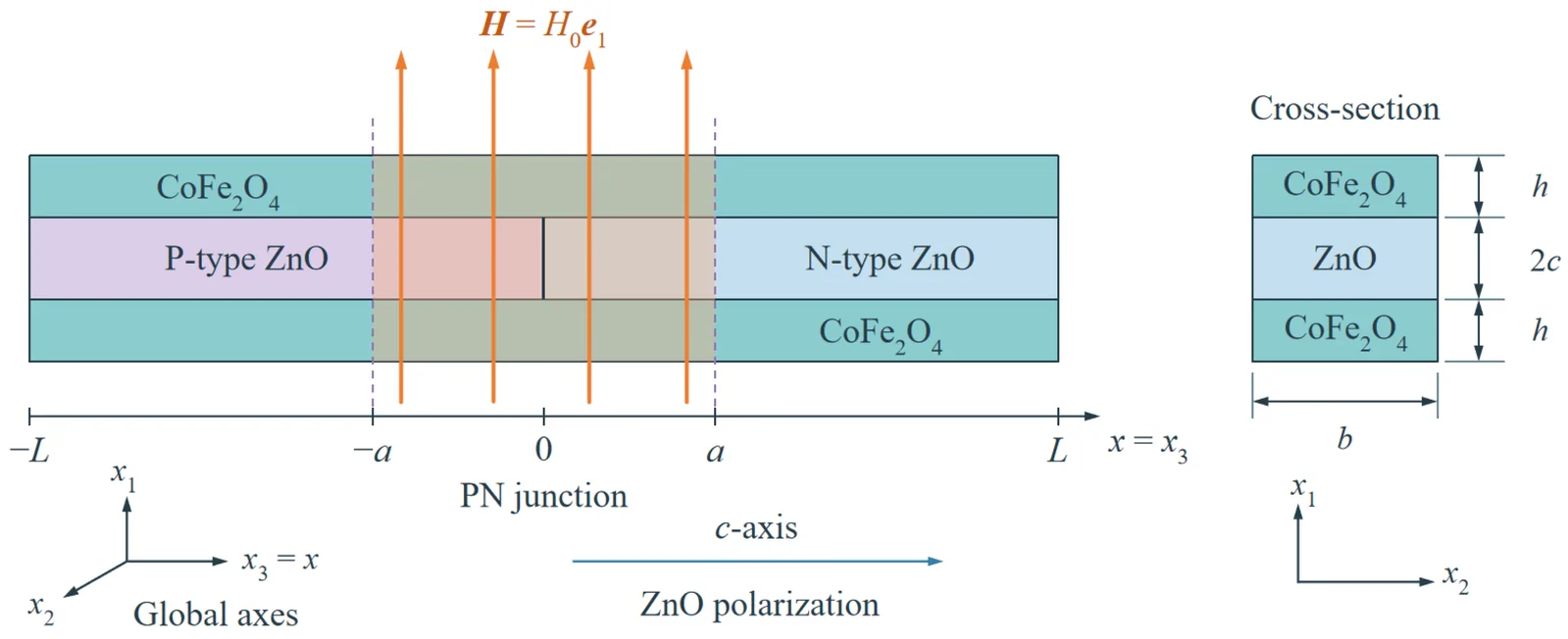
Symmetric piezomagnetic/piezoelectric semiconductor PN-junction/piezomagnetic sandwich fiber under a local transverse magnetic field. The central layer has thickness 2*c*, each piezomagnetic layer has thickness *h*, and the fiber has width *b* and length 2*L*. The P and N regions meet at *x* = 0. The polarization axis is along *x*_3_, and the magnetic field is applied along *x*_1_ over the central region. The cross section is shown to the right of the main view.

Consider the symmetric sandwich fiber of length 2*L* shown in Figure 1. A piezoelectric semiconductor layer is placed between two identical piezomagnetic layers. The axial coordinate is *x* = *x*_3_, while *x*_1_ and *x*_2_ denote the thickness and width directions, respectively. The central layer has thickness 2*c*, each piezomagnetic layer has thickness *h*, and all layers have width *b*. The semiconductor cross-sectional area and the combined area of the two piezomagnetic layers are therefore

*A*_s_ = 2*bc*,   *A*_m_ = 2*bh* (1)

A PN homojunction is formed at *x* = 0 in the semiconductor layer, with the P and N regions occupying *x* < 0 and *x* > 0, respectively. The two regions have identical elastic, piezoelectric, and dielectric coefficients and the same polarization axis along the positive fiber direction; they differ in their reference carrier concentrations. Perfect bonding is assumed. The material orientations and loading of the two piezomagnetic layers are symmetric about the mid-plane, so their axial magnetic driving contributions have the same sign. Within the slender-fiber approximation, a common axial extensional deformation and cross-sectionally averaged electrical fields are retained.

A one-dimensional section average requires both transverse dimensions to be small relative to the axial lengths over which strain, electric field, and magnetic excitation vary, including the magnetic-field transition width. It also requires many mobile carriers and ionized dopants in each representative averaging volume. These conditions are dimensional criteria, not a universal nanometer range. An ideal step profile is a limiting source description; its immediate neighborhood cannot satisfy a strict separation of transverse and axial scales.

The transverse magnetic field is applied along the thickness direction and is written as ***H*** = *H*(*x*)***e***_1_. The half-width of the field-excited region satisfies 0 < *a* < *L*, where *L* is the fiber half-length. Its axial distribution is prescribed as

(2) $$H\left(x\right)=\left\{\begin{array}{ll} H_{0},\; & |x|<a\;\\ 0,\; & a<|x|\leq L \end{array}\right.$$

Here, *H*_0_ denotes a signed magnetic-field increment about a prescribed biased operating state, at which the effective magnetostrictive coefficients are evaluated as local tangent coefficients. Their use does not imply intrinsic zero-bias piezomagnetism of CoFe_2_O_4_. All subsequent fields are defined relative to this state, with the background stress and polarization equilibrated; *H*_0_ = 0 denotes the zero-increment state. The magnetic field is prescribed by its cross-sectional average, without changes in hysteresis, saturation, or demagnetizing fields. The edges of the field-excited region at *x* = ±*a* and the PN doping interface at *x* = 0 are three distinct matching locations. The prescribed increment is the field within the magnetic layers in the one-dimensional model. It is not converted from an externally applied field: that conversion requires a measured magnetization curve, demagnetizing factor, and device geometry. Neither the magnetic bias value nor a thin-film tangent coefficient at that bias is reported by the parameter source used below.

### 2.2. Reference Carrier State

The actual hole and electron concentrations are expressed as *p* = *p*_0_ + Δ*p* and *n* = *n*_0_ + Δ*n*, respectively. The reference concentrations in the P and N regions are denoted by (*p*_0P_, *n*_0P_) and (*p*_0N_, *n*_0N_). Each reference region satisfies local charge neutrality:

(3) $$p_{0r}-n_{0r}+N_{Dr}^{+}-N_{Ar}^{-}=0,\;\;\;r=P,\;N$$

The ionized donor and acceptor concentrations are constant within each doping region. This piecewise neutral state serves as the linearization reference. Both junction formation and the applied magnetic-field increment contribute to the redistribution represented by Δ*p* and Δ*n*. At uniform temperature, with no carrier generation or recombination and with carrier-blocking exterior surfaces, the total numbers of holes and electrons are conserved separately. The axial drift and diffusion currents balance in the steady state. The near-equal electron and hole populations used for the weak-junction calculation are prescribed independently conserved reference populations, not a room-temperature dark-equilibrium ZnO PN junction. A transient optically or electrically prepared ambipolar state would require the carrier redistribution time to be shorter than the recombination time, with observation between those scales. The present model does not specify a carrier-generation mechanism or recombination lifetime; the preparation and duration of such a state therefore remain conditions for its physical realization.

The analytical solution uses the small-carrier-perturbation approximation, subject to

(4) $$\eta_{\mathrm{lin}}=\underset{x,\;r}{\max}\left\{\frac{\left|\Delta p_{r}(x)\right|}{p_{0r}},\;\frac{\left|\Delta n_{r}(x)\right|}{n_{0r}}\right\}\ll 1$$

Equation (4) constrains the total concentration perturbation caused by junction formation and the applied magnetic-field increment. Continuity of the actual concentrations at the PN interface gives the necessary condition *η*_lin_ ≥ max{|*p*_0P_ − *p*_0N_|/(*p*_0P_ + *p*_0N_), |*n*_0P_ − *n*_0N_|/(*n*_0P_ + *n*_0N_)}, while the full-domain perturbation is evaluated from the resulting concentration profiles. Weak-junction reference concentrations are adopted below, and Section 5 examines the linearization error using nonlinear drift–diffusion solutions.

## 3. Governing Equations

### 3.1. Cross-Sectional Reduction and Magnetomechanical Coupling

Let *u*(*x*) and *ϕ*(*x*) denote the cross-sectionally averaged axial displacement and electric potential. The axial strain and electric field are *S* = *u*′ and *E* = −*ϕ*′, respectively, where a prime denotes differentiation with respect to *x*. After transverse-stress relaxation, the constitutive relations of the semiconductor and piezomagnetic layers take the form

(5) $$\begin{array}{cc} T_{s}=c_{s}S-e_{s}E,\; & \;D_{s}=e_{s}S+\varepsilon_{s}E\;\\ T_{m}=c_{m}S-h_{m}H,\; & D_{m}=\varepsilon_{m}E \end{array}$$

The subscripts s and m identify the semiconductor and piezomagnetic phases, respectively. Here, *c*_s_, *c*_m_ are the effective elastic coefficients, *e*_s_ and *h*_m_ are the effective piezoelectric and piezomagnetic coefficients, and *ε*_s_, *ε*_m_ are the axial dielectric coefficients. Magnetoelectric interaction between the phases arises through mechanical compatibility. For a hexagonal piezoelectric semiconductor polarized along the fiber axis, elimination of the transverse stresses gives

(6) $$\begin{array}{c} c_{s}=c_{33}^{s}-\frac{2{\left(c_{13}^{s}\right)}^{2}}{c_{11}^{s}+c_{12}^{s}}\;\\ e_{s}=e_{33}^{s}-\frac{2c_{13}^{s}e_{31}^{s}}{c_{11}^{s}+c_{12}^{s}}\;\\ \varepsilon_{s}=\varepsilon_{33}^{s}+\frac{2{\left(e_{31}^{s}\right)}^{2}}{c_{11}^{s}+c_{12}^{s}} \end{array}$$

The local axes of the piezomagnetic layers are $x_{1}^{\prime }=x_{3}$, $x_{2}^{\prime }=-x_{2}$, and $x_{3}^{\prime }=x_{1}$. Thus, the transverse magnetic field acts along the local $x_{3}^{\prime }$ direction, whereas axial extension occurs along $x_{1}^{\prime }$. With $\Delta_{m}=c_{11}^{m}c_{33}^{m}-{\left(c_{13}^{m}\right)}^{2}$, the corresponding effective coefficients are

(7) $$\begin{array}{ccc} c_{m} & = & c_{11}^{m}-\frac{c_{33}^{m}{\left(c_{12}^{m}\right)}^{2}-2c_{12}^{m}{\left(c_{13}^{m}\right)}^{2}+c_{11}^{m}{\left(c_{13}^{m}\right)}^{2}}{\Delta_{m}}\;\\ h_{m} & = & h_{31}^{m}-\frac{[c_{12}^{m}c_{33}^{m}-{\left(c_{13}^{m}\right)}^{2}]h_{31}^{m}+(c_{11}^{m}-c_{12}^{m})c_{13}^{m}h_{33}^{m}}{\Delta_{m}}\;\\ \varepsilon_{m} & = & \varepsilon_{11}^{m} \end{array}$$

The extensional reduction imposes the phasewise axial compatibility conditions *S*_s_ = *S*_m_ = *S* = *u*′(*x*) and *E*_3s_ = *E*_3m_ = *E*_3_ = −*ϕ*′(*x*). The common electric field is the axial *x*_3_-component tangential to the layer interfaces; the approximation does not impose equality of transverse fields.

The three-dimensional coefficients in Equations (6) and (7) are expressed in the material axes of each phase, giving $\varepsilon_{11}^{m}$ as the axial dielectric coefficient of the piezomagnetic layers. The one-dimensional reduction uses phasewise transverse-stress relaxation, with interlayer constraints represented by a common axial strain and a continuous tangential electric field. Interlayer shear, local end relaxation, and transverse electric fields are neglected. Cross-sectional integration of the constitutive relations gives the axial force resultant *N* and integrated electric displacement *Q*:

(8) $$\begin{array}{ccc} N & = & \mathrm{KS}-\mathrm{PE}-\mathrm{MH}\;\\ Q & = & \mathrm{PS}+\mathrm{BE} \end{array}$$

Here, *K* = *c*_s_*A*_s_ + *c*_m_*A*_m_ is the axial stiffness, *P* = *e*_s_*A*_s_ and *M* = *h*_m_*A*_m_ are cross-sectional coupling coefficients, and *B* = *ε*_s_*A*_s_ + *ε*_m_*A*_m_ is the cross-sectional dielectric coefficient. Mechanical equilibrium and traction-free ends imply a vanishing axial force resultant throughout the fiber, giving

(9) $$S=\mathrm{vE}+\mathrm{wH},\;\;\;v=\frac{P}{K},\;\;w=\frac{M}{K}$$

Substituting Equation (9) into the integrated electric displacement and normalizing by the semiconductor cross-sectional area gives

(10) $$\bar{D}=\frac{Q}{A_{s}}=\varepsilon_{c}E+m_{H}H$$

The effective dielectric coefficient and magnetically induced electric-displacement coefficient are

(11) $$\varepsilon_{c}=\frac{B+P^{2}/K}{A_{s}},\;\;\;m_{H}=\frac{\mathrm{PM}}{KA_{s}}$$

The effective magnetically induced electric-polarization source in Equation (10) arises from piezomagnetic deformation transferred through interlayer mechanical coupling. Both its strength and the effective dielectric coefficient depend on the cross-sectional configuration, so layer thickness affects magnetic driving and carrier screening simultaneously. Although the force resultant vanishes, the phases can sustain self-equilibrated axial stresses. The normalized electric displacement $\bar{D}=Q/A_{s}$ includes contributions from both phases and uses the semiconductor area as its reference, whereas *D*_s_ is the displacement within the semiconductor phase.

### 3.2. Two-Carrier Drift–Diffusion and the Screening Equation

Neglecting the net electric-displacement flux through the outer lateral surface, cross-sectional integration of Gauss’s law gives

(12) $$Q^{\prime }=qA_{s}\left(\Delta p-\Delta n\right),\;\;\;{\bar{D}}^{\prime }=q\left(\Delta p-\Delta n\right)$$

The cross-sectional balance before this electrical approximation is d*D*/d*x* + *f*_lat_ = *qF*, where *f*_lat_ is the outward lateral electric-displacement flux per unit axial length divided by the semiconductor area. Equation (12) sets this term to zero. This assumption is independent of carrier blocking, and its accuracy depends on the exterior dielectric environment and transverse field distribution.

Here, *q* > 0 is the elementary charge. At a uniform reference temperature, the linearized drift–diffusion current densities, together with the Einstein relations, are

(13) $$\begin{array}{ccc} J^{p} & = & q\mu^{p}p_{0}E-qD^{p}\Delta p^{\prime }\;\\ J^{n} & = & q\mu^{n}n_{0}E+qD^{n}\Delta n^{\prime }\;\\ \frac{D^{p}}{\mu^{p}} & = & \frac{D^{n}}{\mu^{n}}=V_{T},\;\;\;V_{T}=\frac{k_{B}T_{0}}{q} \end{array}$$

For the conventional currents in Equation (13), the carrier-continuity equations including pair generation and recombination are ∂*p*/∂*t* = −(1/*q*)(∂*J^p^*/∂*x*) + *G* − *R*, ∂*n*/∂*t* = (1/*q*)(∂*J^n^*/∂*x*) + *G* − *R*, where *G* and *R* are volumetric generation and recombination rates. In the source-free stationary specialization used here, each current is constant along the fiber; blocking at both ends then gives *J^p^* = *J^n^* = 0. Thus, the zero-current relations follow from continuity and the end conditions.

A transient realization of the prescribed two-carrier state would require *τ*_tr_ ≪ *t*_obs_ ≪ *τ*_rec_, where the transport-relaxation time, observation time, and recombination time are denoted by the three quantities, respectively. Transport must have relaxed while the independently conserved carrier populations remain approximately unchanged. The present stationary equations supply no lifetime or preparation mechanism, so this condition is an applicability requirement rather than a demonstrated time window. Continuous optical or electrical pumping would require explicit generation, recombination, and injection terms.

The quantities *μ^p^*, *μ^n^*, *D^p^*, *D^n^*, *k*_B_, and *V*_T_ denote the mobilities, diffusion coefficients, Boltzmann constant, and thermal voltage, respectively. Steady-state continuity and blocking end conditions give *J^p^* = *J^n^* = 0, and hence Δ*p*′ = (*p*_0_/*V*_T_)*E* and Δ*n*′ = −(*n*_0_/*V*_T_)*E*. To treat both carrier species, define the net concentration perturbation and screening parameter as

(14) $$F_{r}=\Delta p_{r}-\Delta n_{r},\;\;\;\gamma_{r}=\frac{V_{T}}{p_{0r}+n_{0r}},\;\;\;k_{r}^{2}=\frac{q}{\varepsilon_{c}\gamma_{r}}$$

Substitution of the zero-current relations into Equation (12), together with Equation (10), gives the reduced equations within each uniformly doped region:

(15) $$E_{r}=\gamma_{r}F_{r}^{\prime },\;\;\;F_{r}^{\prime \prime }-k_{r}^{2}F_{r}=-\frac{m_{H}}{\varepsilon_{c}\gamma_{r}}H^{\prime }$$

The screening length is *l*_s,*r*_ = 1/*k_r_*. Equation (15) shows that a spatially varying magnetically induced electric polarization drives carrier redistribution through the magnetic-field gradient. The equation holds within regions of constant reference concentrations; where the magnetic field is constant, the net perturbation satisfies a homogeneous screening equation. For a stepwise field, the distributional contribution of its gradient is incorporated through continuity of electric displacement at the magnetic boundaries. Reference-concentration differences at the PN interface are instead handled by matching the actual carrier concentrations.

Defining *η_pr_* = *p*_0*r*_/(*p*_0*r*_ + *n*_0*r*_), *η_nr_* = *n*_0*r*_/(*p*_0*r*_ + *n*_0*r*_) and integrating the zero-current relations gives

(16) $$\begin{array}{ccc} \phi_{r} & = & -\gamma_{r}F_{r}+C_{\mathrm{\phi r}},\;\\ \Delta p_{r} & = & \eta_{\mathrm{pr}}F_{r}+C_{cr},\;\\ \Delta n_{r} & = & -\eta_{\mathrm{nr}}F_{r}+C_{cr} \end{array}$$

*C_ϕr_* are the potential integration constants on the two sides, and *C*_c*r*_ are determined by concentration continuity and carrier conservation. The common additive constant in the two concentration expressions follows from *F_r_* = Δ*p_r_* − Δ*n_r_*. The zero-flux conditions and Einstein relations eliminate the mobilities and diffusion coefficients, leaving the steady distributions governed by the reference concentrations, thermal voltage, and effective dielectric coefficient.

### 3.3. Nonlinear Reference Problem and Dimensionless Variables

To assess drift linearization, the actual carrier concentrations are retained while keeping the same one-dimensional constitutive relations, doping distribution, and end conditions. The zero-current equations and Gauss’s law then form the nonlinear reference system

(17) $$\begin{array}{ccc} p^{\prime } & = & \frac{\mathrm{pE}}{V_{T}},\;\;\;n^{\prime }=-\frac{\mathrm{nE}}{V_{T}}\\ \varepsilon_{c}E^{\prime }+m_{H}H^{\prime } & = & q(p-n+n_{0}-p_{0}) \end{array}$$

Equation (17) is solved in the four subregions with the same end conditions, interface conditions, and carrier conservation constraints as the linear model. The zero-flux relations give *p* = *C_p_*exp(−*ϕ*/*V*_T_) and *n* = *C_n_*exp(*ϕ*/*V*_T_); hence, *pn* = *C_p_C_n_* is constant, with its value determined by the total hole and electron populations. Introduce the following dimensionless variables:

(18) $$\hat{x}=\frac{x}{L},\;\;\hat{\phi }=\frac{\phi }{V_{T}},\;\;\hat{E}=\frac{\mathrm{EL}}{V_{T}},\;\;\hat{p}=\frac{p}{N_{*}},\;\;\hat{n}=\frac{n}{N_{*}}$$

The associated concentration scale, magnetic amplitude, and dimensionless electric displacement are

(19) $$N_{*}=\frac{\varepsilon_{c}V_{T}}{qL^{2}},\;\;\;A=\frac{m_{H}H_{0}L}{\varepsilon_{c}V_{T}},\;\;\;\hat{\bar{D}}=\frac{\bar{D}L}{\varepsilon_{c}V_{T}}$$

The scales in Equations (18) and (19) use *ε*_c_ for the current structure; thus, N_*_ and *A* vary with the effective dielectric coefficient as the layer thickness changes. The layer-thickness comparison can equivalently use the coefficient *ε*_ref_ at *r* = 1 and the fixed concentration scale $N_{*,\mathrm{ref}}=\varepsilon_{\mathrm{ref}}V_{T}/qL^{2}$, with the ratio *ε*_c_(*r*)/*ε*_ref_ retained in the dimensionless equations. Accordingly, *A* varies during a scan at fixed physical reference concentrations and applied magnetic field. The dimensionless magnetic amplitude is denoted consistently by *A* in the figures.

## 4. Analytical Solution Under a Local Magnetic Field

### 4.1. Boundary and Continuity Conditions

The magnetic boundaries *x* = −*a*, *a* and the doping interface *x* = 0 divide the fiber into four subregions: (−*L*, −*a*), (−*a*, 0), (0, *a*), and (*a*, *L*). The first two use the P-region reference concentrations and the last two use the N-region values. The fiber ends are mechanically traction-free, electrically open, and blocking to carriers:

(20) $$N\left(\pm L\right)=0,\;\;\;\bar{D}\left(\pm L\right)=0,\;\;\;J^{p}\left(\pm L\right)=J^{n}\left(\pm L\right)=0$$

Equation (20) imposes two distinct electrical conditions. Carrier-blocking ends give *J^p^* = *J^n^* = 0, whereas *D*(±*L*) = 0 additionally assumes no prescribed free end charge and a negligible external axial displacement field. Both ends lie outside the field-excited region. The axial displacement and electric potential are referenced to their junction-center values, and global hole conservation is imposed as

(21) $$\phi \left(0\right)=0,\;\;\;u\left(0\right)=0,\;\;\;\int_{-L}^{L}\Delta p\;dx=0$$

Integrating Equation (12) along the fiber and using zero electric displacement at both ends gives ∫−LLF dx=0. Hole conservation and the integrated Gauss equation therefore also ensure conservation of the total electron population.

Let ${\left[g\right]}_{x_{i}}=g(x_{i}^{+})-g(x_{i}^{-})$ denote the jump at an interface, where *x_i_* = −*a*, 0, *a*. At an ideal semiconductor homojunction with no traps, additional interface barrier, or free surface charge, the displacement, potential, electric displacement, and actual carrier concentrations satisfy

(22) $${\left[u\right]}_{x_{i}}={\left[\phi \right]}_{x_{i}}={\left[\bar{D}\right]}_{x_{i}}={\left[p\right]}_{x_{i}}={\left[n\right]}_{x_{i}}=0$$

The force resultant and both carrier currents vanish throughout the fiber, so their continuity is automatic. Substituting Equations (10) and (16) into Equation (22) gives six matching relations for the net carrier perturbation:

(23) $$\begin{array}{ccc} {\left[F\right]}_{-a} & = & {\left[F\right]}_{a}=0\;\\ \varepsilon_{c}\gamma_{P}{\left[F^{\prime }\right]}_{-a} & = & -m_{H}H_{0}\;\\ \varepsilon_{c}\gamma_{N}{\left[F^{\prime }\right]}_{a} & = & m_{H}H_{0}\;\\ F(0^{-})-F(0^{+}) & = & J\;\\ \gamma_{P}F^{\prime }(0^{-}) & = & \gamma_{N}F^{\prime }(0^{+}) \end{array}$$

Here, J = (*p*_0N_ − *p*_0P_) − (*n*_0N_ − *n*_0P_) is the mismatch between the reference net carrier concentrations on the two sides. At a magnetic boundary, *F* is continuous while *F*’ jumps. At the central PN interface, *F* jumps while *γF*’ = *E* remains continuous. These distinct conditions enforce matching of the actual concentrations and electric displacement at the corresponding interfaces.

### 4.2. Piecewise Field Solutions and Integration Constants

Within each constant-field region, Equation (15) has a general solution in hyperbolic functions. Define *d_r_* = *ε*_c_*γ_r_* and *s_r_* = *m_H_H*_0_/*d_r_*. Applying the two end conditions and the four magnetic-boundary conditions reduces the eight homogeneous integration constants to two unknown coefficients:

(24) $$F\left(x\right)=\left\{\begin{array}{ll} U_{P}\cosh[k_{P}(x+L)],\; & -L\leq x<-a\;\\ U_{P}\cosh[k_{P}(x+L)]-\frac{s_{P}}{k_{P}}\sinh[k_{P}(x+a)],\; & -a\leq x<0\;\\ U_{N}\cosh[k_{N}(L-x)]+\frac{s_{N}}{k_{N}}\sinh[k_{N}(a-x)],\; & 0<x\leq a\;\\ U_{N}\cosh[k_{N}(L-x)],\; & a<x\leq L \end{array}\right.$$

The first term in each branch of Equation (24) satisfies the zero-derivative condition at the corresponding free end. The additional hyperbolic-sine term within the field-excited region accounts explicitly for the derivative jump at the magnetic boundary. To determine *U*_P_, *U*_N_, set *C_r_* = cosh(*k_r_L*) and *T_r_* = *d_r_k_r_*sinh(*k_r_L*), and define

(25) $$\begin{array}{ccc} R & = & J+\frac{s_{P}}{k_{P}}\sinh(k_{P}a)+\frac{s_{N}}{k_{N}}\sinh(k_{N}a)\;\\ V & = & m_{H}H_{0}[\cosh(k_{P}a)-\cosh(k_{N}a)]\;\\ D & = & C_{P}T_{N}+C_{N}T_{P} \end{array}$$

The quantities R, V, D are algebraic combinations used to solve for the integration constants; D is the positive denominator of the resulting two-equation matching system. Substitution of Equation (24) into the two independent PN-interface conditions yields

(26) $$U_{P}=\frac{RT_{N}+C_{N}V}{D},\;\;\;U_{N}=\frac{C_{P}V-T_{P}R}{D}$$

For positive effective permittivity, reference concentrations, and fiber length, the denominator in Equation (25) is positive, and Equation (26) has a unique solution. The concentration-mismatch term produces the baseline junction response, while the magnetic term produces the additional response. The eight net-perturbation integration constants are determined by two end conditions, four magnetic-boundary conditions, and two PN-interface conditions. Hole continuity and total hole conservation determine the two additional concentration constants. Electron continuity and conservation follow from the net-perturbation jump and the integrated Gauss equation, respectively.

To determine the concentration constants, denote the one-sided junction values by *f*_P_ = *U*_P_*C*_P_ − (*s*_P_/*k*_P_)sinh(*k*_P_*a*) and *f*_N_ = *U*_N_*C*_N_ + (*s*_N_/*k*_N_)sinh(*k*_N_*a*), and integrate the net perturbation over each half of the fiber:

(27) $$\begin{array}{c} I_{P}=\int_{-L}^{0}F\;dx=\frac{U_{P}}{k_{P}}\sinh(k_{P}L)-\frac{s_{P}}{k_{P}^{2}}[\cosh(k_{P}a)-1]\;\\ I_{N}=\int_{0}^{L}F\;dx=\frac{U_{N}}{k_{N}}\sinh(k_{N}L)+\frac{s_{N}}{k_{N}^{2}}[\cosh(k_{N}a)-1] \end{array}$$

Hole continuity gives *C*_cN_ − *C*_cP_ = *η_p_*_P_*f*_P_ − *η_p_*_N_*f*_N_ − (*p*_0N_ − *p*_0P_) ≡ *W*. Combining this relation with global conservation in Equation (21) yields

(28) $$C_{\mathrm{cP}}=-\frac{\eta_{pP}I_{P}+\eta_{pN}I_{N}+\mathrm{LW}}{2L},\;\;\;C_{\mathrm{cN}}=C_{\mathrm{cP}}+W$$

With the concentration constants determined and both displacement and potential referenced to zero at the junction center, the electrical fields and axial displacement are

(29) $$\begin{array}{ccc} E_{r}(x) & = & \gamma_{r}F_{r}^{\prime }(x)\\ \phi_{r}(x) & = & -\gamma_{r}[F_{r}(x)-f_{r}]\;\\ \Delta p_{r}(x) & = & \eta_{\mathrm{pr}}F_{r}(x)+C_{cr}\;\\ \Delta n_{r}(x) & = & -\eta_{\mathrm{nr}}F_{r}(x)+C_{cr}\;\\ {\bar{D}}_{r}(x) & = & \varepsilon_{c}E_{r}(x)+m_{H}H(x)\;\\ u(x) & = & -\mathrm{v\phi}(x)+w\int_{0}^{x}H(\xi )\;d\xi \end{array}$$

Here, *r* = *P*, *N*, and *F_r_* takes the corresponding branch of Equation (24) in each subregion. The magnetic-field integral is $\int_{0}^{x}H(\xi )d\xi =H_{0}\mathrm{sgn}(x)\min(|x|,\;a)$. Equations (24), (26), (28) and (29) specify the electrical field and displacement in all four regions. The electric field and strain jump at the magnetic boundaries, whereas displacement, potential, electric displacement, and actual concentrations remain continuous; the axial phase stresses are self-equilibrated. At the PN interface, the one-sided net-perturbation values *F*(0^±^) compensate the reference-concentration steps, preserving continuity of the actual concentrations.

### 4.3. Junction Field and Spatial Screening

For a fixed structure and fixed reference concentrations, the analytical problem is linear. Each field can therefore be separated into a zero-increment PN-junction baseline and a magnetically induced contribution:

*ϕ* = *ϕ*_PN_ + *ϕ_H_*,   *E* = *E*_PN_ + *E_H_*,   *F* = *F*_PN_ + *F_H_* (30)

The baseline is the solution for the same sandwich structure at *H*_0_ = 0, retaining the piezomagnetic layers’ contributions to stiffness and dielectric response. To characterize transfer from the magnetic boundaries to the junction center, introduce

(31) $$\alpha_{H}=\frac{m_{H}}{\varepsilon_{c}},\;\;\;Y_{r}=\frac{\coth(k_{r}L)}{\gamma_{r}k_{r}},\;\;\;R_{r}=\frac{\cosh[k_{r}(L-a)]}{\cosh(k_{r}L)}$$

Differentiating Equation (24) at the junction center and using Equation (26) gives

(32) $$E\left(0\right)=\frac{J}{Y_{P}+Y_{N}}-\alpha_{H}H_{0}\frac{Y_{P}R_{P}+Y_{N}R_{N}}{Y_{P}+Y_{N}}$$

The first term in Equation (32) is the center field caused by the reference-concentration mismatch. The second is the magnetically induced electric-field contribution attenuated by carrier screening. Equating their sum to zero gives the magnetic amplitude required for center-field compensation:

(33) $$H_{\mathrm{comp}}=\frac{\varepsilon_{c}J}{m_{H}(Y_{P}R_{P}+Y_{N}R_{N})}$$

For *m_H_* ≠ 0, Equation (33) gives the direction and amplitude of the compensation field, with the associated concentration perturbation evaluated using Equation (4). Equal total reference concentrations on the two sides give *k*_P_ = *k*_N_ = *k* and *γ*_P_ = *γ*_N_ = *γ*, reducing the junction-center field to

(34) $$\begin{array}{ccc} E_{\mathrm{PN}}(0) & = & \frac{\mathrm{\gamma kJ}}{2}\tanh(\mathrm{kL})\;\\ E_{H}(0) & = & -\alpha_{H}H_{0}R,\;\;\;R=\frac{\cosh[k(L-a)]}{\cosh(\mathrm{kL})} \end{array}$$

*R* is the junction-center field transfer coefficient normalized by the magnetic driving scale. Set *ϕ_H_*(0) = 0 and define the local magnetically induced electric-potential amplitude *Φ_H_* = max|*ϕ_H_*| and the magnetically induced end-to-end potential difference *V_H_* = *ϕ_H_*(*L*) − *ϕ_H_*(−*L*). For *m_H_H*_0_ ≠ 0, their normalized relations are

(35) $$\begin{array}{ccc} \Phi_{H} & = & \frac{\left|\alpha_{H}H_{0}\right|}{k}\sinh(\mathrm{ka})R\;\\ V_{H} & = & \frac{2\alpha_{H}H_{0}}{k}\frac{\sinh(\mathrm{ka})}{\cosh(\mathrm{kL})}\;\\ \frac{\left|V_{H}\right|}{2\Phi_{H}} & = & \mathrm{sech}[k(L-a)] \end{array}$$

Under symmetric screening, the extrema of the magnetically induced electric potential occur at *x* = ±*a*. Equation (35) also accounts for screening between each field-region edge and the adjacent fiber end, thereby distinguishing the local extrema from the end-to-end potential difference. If *k*(*L* − *a*) ≫ 1, end corrections are small, and the transfer coefficient and normalized local-potential amplitude satisfy

(36) $$R≃e^{-\mathrm{ka}},\;\;\;P_{\phi }=\frac{2k\Phi_{H}}{\left|\alpha_{H}H_{0}\right|}≃1-e^{-2\mathrm{ka}},\;\;\;P_{\phi }+R^{2}≃1$$

Equation (36) gives different loading-width dependences for the boundary potential and the center field: the former increases, whereas the latter decays with increasing screening distance. For *ka* ≫ 1 and *k*(*L* − *a*) ≫ 1, the local potential approaches |*α_H_H*_0_|/(2*k*). End corrections grow as the field-region edges approach the fiber ends; Equation (35) gives the limiting local amplitude |*α_H_H*_0_|tanh(*kL*)/*k* as *a* → *L*^−^.

To quantify regulation over a finite junction region, choose a fixed observation interval [−l,l] and define its root-mean-square electric field as

(37) $$E_{J}\left(H_{0}\right)={\left[\frac{1}{2l}\int_{-l}^{l}E^{2}(x;H_{0})\;dx\right]}^{1/2}$$

Here, l is the fixed evaluation half-width satisfying 0 < l < *L*, while *L* and *a* are the fiber half-length and magnetic-region half-width, respectively. Denoting the electric field per unit magnetic increment by *g_H_*(*x*) = ∂*E*/∂*H*_0_ gives *E*(*x*;*H*_0_) = *E*_PN_(*x*) + *H*_0_*g_H_*(*x*). Minimizing the integrated squared field in Equation (37) yields

(38) $$H_{\mathrm{RMS}}=-\frac{\int_{-l}^{l}E_{\mathrm{PN}}(x)g_{H}(x)\;dx}{\int_{-l}^{l}g_{H}^{2}(x)\;dx}$$

For $\int_{-l}^{l}g_{H}^{2}\;dx>0$, Equation (38) uniquely determines the magnetic amplitude that minimizes the regional root-mean-square field. This amplitude depends on both field profiles throughout the evaluation interval and approaches the center-compensating value as l → 0, provided *g_H_*(0) ≠ 0. With loading or concentration-perturbation constraints, the minimum is taken over the corresponding admissible interval.

### 4.4. Layer Thickness and Magnetic Transition Width

Let the thickness ratio be *r* = *h*/*c* = *A*_m_/*A*_s_. At fixed total thickness, fiber length, physical reference concentrations, and applied magnetic field, Equation (11) gives

(39) $$\begin{array}{ccc} \varepsilon_{c}(r) & = & \varepsilon_{s}+\varepsilon_{m}r+\frac{e_{s}^{2}}{c_{s}+c_{m}r}\;\\ m_{H}(r) & = & \frac{e_{s}h_{m}r}{c_{s}+c_{m}r}\\ \alpha_{H}(r) & = & \frac{e_{s}h_{m}r}{(\varepsilon_{s}+\varepsilon_{m}r)(c_{s}+c_{m}r)+e_{s}^{2}} \end{array}$$

The boundary electric-field jump has magnitude |*α_H_H*_0_|. For positive effective stiffness and dielectric coefficients and nonzero magnetomechanical coupling, maximizing this magnitude with respect to the thickness ratio gives

(40) $$r_{\mathrm{jump}}=\sqrt{\frac{\varepsilon_{s}c_{s}+e_{s}^{2}}{\varepsilon_{m}c_{m}}}$$

By contrast, optimization of the junction-center field and local potential involves |*α_H_*(*r*)|*R*[*k*(*r*)], |*α_H_*(*r*)|/*k*(*r*)sinh[*k*(*r*)*a*]*R*[*k*(*r*)], respectively. Both depend on $k(r)\propto {\left[\varepsilon_{c}(r)\right]}^{-1/2}$ as well as on the magnetoelectric conversion coefficient, so their optimal thickness ratios generally differ from Equation (40).

A physical magnetic-field boundary has a finite transition width. To quantify this effect, replace the step by a linear transition:

(41) $$H_{\delta }\left(x\right)=\left\{\begin{array}{ll} H_{0},\; & |x|\leq a-\delta /2\;\\ H_{0}\frac{a+\delta /2-|x|}{\delta },\; & a-\delta /2<|x|<a+\delta /2\;\\ 0,\; & |x|\geq a+\delta /2 \end{array}\right.$$

The restriction 0 < *δ* < 2min(*a*, *L* − *a*) prevents the transition from crossing the PN interface or a fiber end. This field has the same peak and axial integral ∫−LLHδ dx=2aH0 as Equation (2). It can be represented as an average of step fields whose half-widths vary uniformly between *a* − *δ*/2 and *a* + *δ*/2. Averaging Equation (34) under symmetric screening gives

(42) $$\frac{E_{H,\delta }(0)}{E_{H,\mathrm{step}}(0)}=\frac{\sinh(k\delta /2)}{k\delta /2}=1+\frac{{\left(k\delta \right)}^{2}}{24}+O\left[{\left(k\delta \right)}^{4}\right]$$

Equation (42) gives the exact center-field correction for the trapezoidal profile relative to the step field, with its second-order expansion valid for *kδ* ≪ 1. At fixed peak amplitude and axial integral, the linear transition distributes the polarization gradient over both sides of the original boundary. Because the screening weight decays nonlinearly with distance, the increased contribution from the part closer to the junction exceeds the reduction from the more distant part, enhancing the magnetically induced electric field at the junction center.

### 4.5. Potential-Difference Response at Fixed Readout Positions

An internal electric-potential difference is a more direct model output for magnetic readout than a single-point field. For two observation positions fixed on a given fiber, subtract the zero-increment reference difference from the difference at a magnetic-field increment to obtain

Δ*V*_12_(*H*_0_) = [*φ*(*x*_2_;*H*_0_) − *φ*(*x*_1_;*H*_0_)] − [*φ*(*x*_2_; 0) − *φ*(*x*_1_; 0)] (43)

Differentiation with respect to the magnetic-field increment defines the potential-difference response coefficient. Its units are V·m·A^−1^. This coefficient describes the internal electrostatic response and is not a calibrated voltage sensitivity of a contacted device.

(44) $$S_{V,H}(x_{1},x_{2})=\frac{\partial {\Delta V}_{12}}{\partial H_{0}}$$

For equal total reference concentrations on the two sides of the junction, the magnetic increment has the same screening parameter in both regions. Let the positive half-spacing of a symmetric fixed pair be *ξ*. The magnetic part of the piecewise solution then gives the following two branches:

*S_V_*_,*H*_(−*ξ*, *ξ*) = ((2*m_H_*)/(*ε*_c_*k*))((cosh[*k*(*L* − *a*)]sinh(*kξ*))/(cosh(*kL*))), 0 ≤ *ξ* ≤ *a* (45)

*S_V_*_,*H*_(−*ξ*, *ξ*) = ((2*m_H_*)/(*ε*_c_*k*))((sinh(*ka*)cosh[*k*(*L* − *ξ*)])/(cosh(*kL*))), *a* ≤ *ξ* ≤ *L* (46)

The inner branch applies when the readout pair lies within the field-excited region; the outer branch applies when the pair lies beyond the field-region edges. The two expressions coincide at their common limit. For fixed material constants and a fixed interior pair, the outer branch increases with loading half-width, whereas the inner branch decreases. The response magnitude is therefore maximized when the field-region edge coincides with the readout positions, as given by

*a*_opt_(*ξ*) = *ξ*, 0 < *ξ* < *L* (47)

This optimum concerns an interior pair and variation of the source width, not a material thickness optimum. The end pair instead follows the outer expression throughout the admissible width range and recovers Equation (35). For asymmetric screening, Equations (43) and (44) remain valid definitions, but the response must be calculated from the four-region solution rather than Equations (45)–(47).

When the loading half-width is varied, the observation positions must remain fixed to represent one readout geometry. If the pair instead follows the moving magnetic boundaries, the comparison involves different layouts.

### 4.6. Equilibrium-State Comparison

A separate equilibrium-state comparison is introduced to determine whether the spatial readout mechanism requires the prescribed, nearly equal carrier populations of the closed weak-junction model. It is not a re-interpretation of those populations as a room-temperature equilibrium state. The mechanical reduction and internal magnetic-increment convention are retained, while the carrier closure is changed explicitly. A symmetric, fully ionized homojunction is considered, with one common equilibrium Fermi level and Maxwell–Boltzmann statistics.

(48) $$n_{\mathrm{eq}}=n_{i}\exp(\psi_{\mathrm{eq}}),p_{\mathrm{eq}}=n_{i}\exp(-\psi_{\mathrm{eq}}),\psi_{\mathrm{eq}}=\frac{\varphi_{\mathrm{eq}}}{V_{T}}$$

Here, the subscript eq denotes the equilibrium reference state, and the intrinsic carrier concentration is determined by the band gap, the conduction- and valence-band effective densities of states, and temperature. The potential at the junction center is set to zero. Symmetric acceptor and donor distributions give an odd potential, which fixes the common-Fermi-level reference. The reference potential is obtained from the nonlinear Poisson equation rather than from the weak-junction solution.

(49) $$-\varepsilon_{c}{\varphi_{\mathrm{eq}}}^{\prime \prime }=q(p_{\mathrm{eq}}-n_{\mathrm{eq}}+{N_{D}}^{+}-{N_{A}}^{-})$$

Differentiating this equilibrium problem with respect to the internal magnetic increment gives the tangent equation below. Its screening coefficient varies through the junction because it depends on the local equilibrium carrier sum. The magnetic source is the same mechanically reduced electric-displacement source as in the analytical model. The comparison assumes unchanged band parameters and fixed ionized dopant charges during the magnetic increment.

(50) $$-\varepsilon_{c}{\delta \varphi }^{\prime \prime }+\frac{q(n_{\mathrm{eq}}+p_{\mathrm{eq}})}{V_{T}}\delta \varphi =-{\left(m_{H}H\right)}^{\prime }$$

The electrostatic termination remains zero end electric displacement, and the applied magnetic region does not reach the ends. Symmetry and the common equilibrium Fermi level make the incremental potential odd. These conditions give the following potential reference and electrical boundary conditions. There is no separate conservation constraint on the total electron and hole populations in this equilibrium comparison.

*δφ*(0) = 0, *δD*(±*L*) = 0, *δD* = −*ε*_c_*δφ*′ + *m_H_H* (51)

At each equilibrium state, the electron-hole product equals the squared intrinsic concentration. Drift and diffusion therefore cancel separately for each carrier, and a detailed-balance generation-recombination law has zero net rate. Electron and hole numbers may change by equal amounts between equilibrium states, while total charge is conserved. This is a quasistatic equilibrium comparison, not a prediction of the time required to reach equilibrium. It neither specifies a photogeneration mechanism nor establishes the fabrication of a p-type ZnO layer.

## 5. Results and Discussion

### 5.1. Material Parameters and Numerical Comparisons

The constitutive parameters are taken for ZnO and CoFe_2_O_4_. For ZnO, the elastic coefficients are $c_{11}^{s}$ = 210 GPa, $c_{12}^{s}$ = 121 GPa, $c_{13}^{s}$ = 105 GPa, and $c_{33}^{s}$ = 211 GPa; the piezoelectric coefficients are $e_{31}^{s}$ = −0.57 C/m^2^ and $e_{33}^{s}$ = 1.32 C/m^2^; and the dielectric coefficient is $\varepsilon_{33}^{s}$ = 8.85 × 10^−11^ F/m [37,43]. For CoFe_2_O_4_, the elastic coefficients are $c_{11}^{m}$ = 286 GPa, $c_{12}^{m}$ = 173 GPa, $c_{13}^{m}$ = 170.5 GPa, and $c_{33}^{m}$ = 269.5 GPa. The piezomagnetic coefficients are $h_{31}^{m}$ = 580.3 N/(A·m) and $h_{33}^{m}$ = 699.7 N/(A·m), and the axial reduction uses $\varepsilon_{11}^{m}$ = 8.00 × 10^−11^ F/m [45]. All coefficients refer to the local material axes. The piezomagnetic coefficients *q*_31_ and *q*_33_ in Ref. [45] are denoted by *h*_31_ and *h*_33_, respectively, in the present notation. The CoFe_2_O_4_ coefficients quoted from Ref. [45] are tabulated constitutive inputs, not measurements of the tangent magnetostrictive coefficient of the proposed thin coatings at a reported bias. Accordingly, the numerical magnetic response is an illustrative small-signal parameter study; its conversion to a specified external bias or to a fabricated film has not been calibrated.

Equations (6) and (7) give *c*_s_ = 1.444 × 10^11^ Pa, *e*_s_ = 1.682 C/m^2^, and *ε*_s_ = 9.046 × 10^−11^ F/m for the semiconductor, and *c*_m_ = 1.543 × 10^11^ Pa, *h*_m_ = 87.31 N/(A · m), and *ε*_m_ = 8.00 × 10^−11^ F/m for the piezomagnetic phase. Taking *c* = 15 nm and *h* = 35 nm gives *r* = 7/3, with effective cross-sectional parameters *ε*_c_ = 2.827 × 10^−10^ F/m, *m_H_* = 6.791 × 10^−10^ C/(A · m), and *α_H_* = 2.402 V/A. With *L* = 4 μm, *a* = 1 μm, *b* = 100 nm, and *T*_0_ = 298 K, the remaining scales are *V*_T_ = 0.02568 V and N_*_ = 2.832 × 10^18^ m^−3^.

Unless otherwise specified, the parameters are *a*/*L* = 0.25, *kL* = 8, *A* = −0.16, and $p_{0P},\;n_{0P},\;p_{0N},\;n_{0N}/N_{*}=\left(32.32,\;31.68,\;31.68,\;32.32\right)$. The total reference concentration on each side is 1.813 × 10^20^ m^−3^, and the baseline magnetic increment is *H*_0_ = −427.7 A/m. The ZnO/CoFe_2_O_4_ parameters specify the electromechanical coefficients, while the carrier concentrations represent a weak-junction reference state with separately conserved populations rather than a dark thermal-equilibrium junction in ZnO at room temperature. The material ratios used in Figure 9 are specified separately. Potential, electric field, and electric displacement below are expressed as $\hat{\phi }=\phi /V_{T}$, $\hat{E}=\mathrm{EL}/V_{T}$, and $\hat{\bar{D}}=\bar{D}L/\varepsilon_{c}V_{T}$, respectively, unless physical units are stated. The physical-unit mapping is an illustrative continuum case; its carrier-count and scaling restrictions are quantified in Section 5.6.

**Figure 2:**
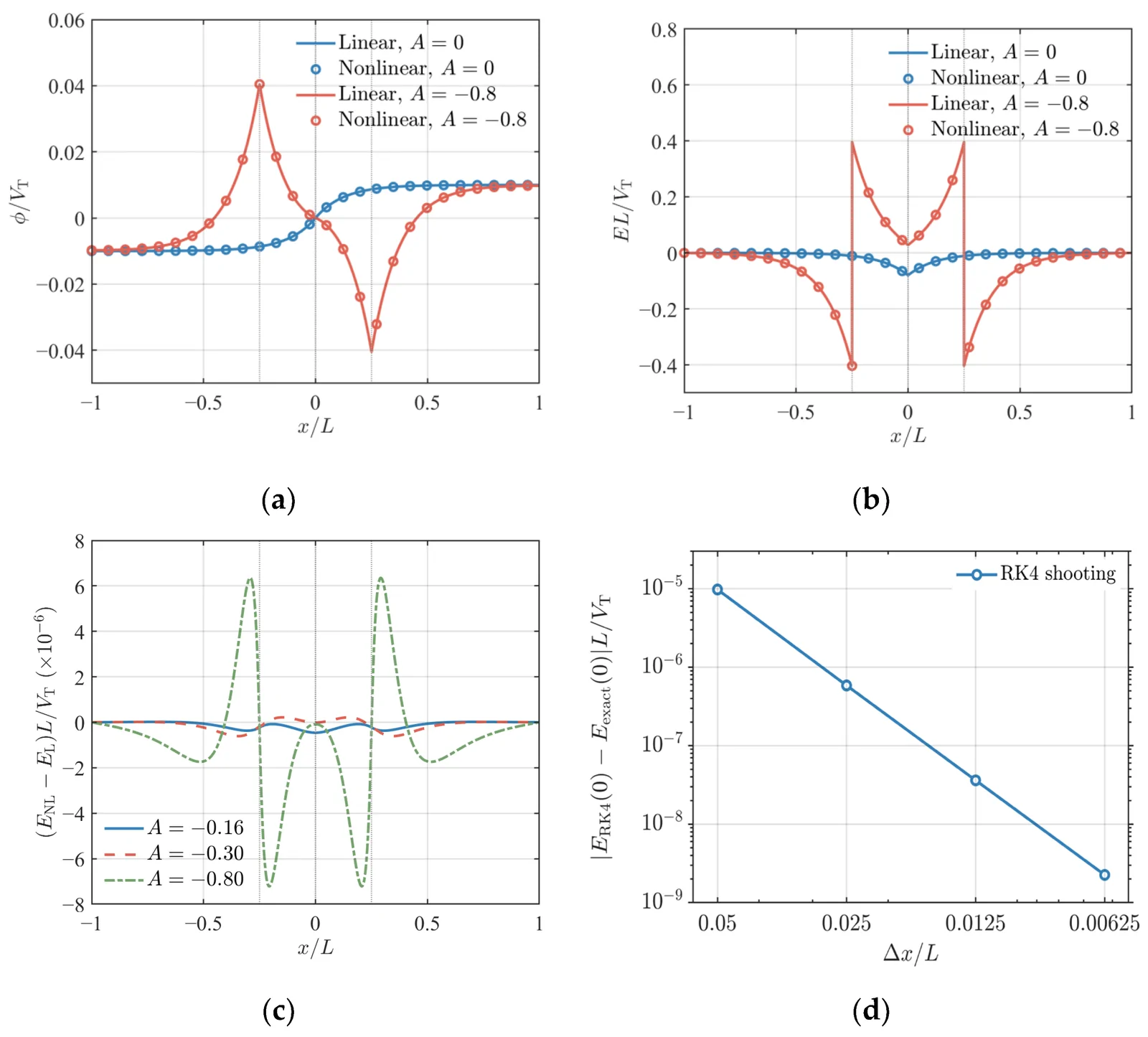
Comparison of analytical and independent numerical solutions: (**a**) electric potential; (**b**) electric field; (**c**) difference between nonlinear and linear electric fields; and (**d**) step-size convergence of fourth-order Runge–Kutta integration. Solid curves denote the linear analytical solution; open circles denote the nonlinear numerical solution in (**a**,**b**). *A* = (*m_H_H*_0_*L*)/(*ε*_c_*V*_T_) is the signed dimensionless magnetic-field increment defined in Equation (19); *A* = 0 is the zero-increment PN reference. Dotted vertical lines indicate the PN interface and the field-region edges. Panel (**c**) compares *A* = −0.16, −0.30, and −0.80.

For the linear problem, the state (*F*, *E*) is integrated in each subregion using the classical fourth-order Runge–Kutta method. Equation (23) is imposed at the interfaces, and the terminal zero-field condition determines the initial value. The nonlinear solutions in Figure 2a–c are obtained by piecewise shooting and cross-checked with a collocation boundary-value solver. Each subregion is mapped to a unit interval with 33 initial nodes and adaptively refined at a relative tolerance of 10^−8^. Actual-concentration continuity, electric-field jumps, end conditions, and total hole conservation are imposed simultaneously. Linear and nonlinear solutions are compared at 501 uniformly spaced points per subregion, using matching one-sided limits at interfaces. Relative error is defined as the maximum electric-field difference divided by the maximum linear-field magnitude.

The finite-element formulation uses continuous electric displacement as the primary unknown, with one-dimensional continuous piecewise-linear elements and three-point Gauss quadrature. Nodes are placed at all interfaces. Magnetically induced electric-displacement sources are integrated by subregion, and the derivative jump at the PN interface is imposed as an interface load. The electric field is recovered by subtracting the magnetically induced electric-displacement term from the discrete electric displacement and dividing by the effective dielectric coefficient. The step-field checks use the five amplitudes compared with the nonlinear solutions, two additional half-widths (one eighth and three eighths of the fiber half-length), concentration multipliers of 0.25 and 4, and the most asymmetric state examined in the doping-asymmetry comparison. The three linear-transition checks use the profiles the trapezoidal magnetic profiles with dimensionless transition widths 0.5, 1, and 2 with equal, spatially uniform hole and electron reference concentrations of 32 times the concentration scale in Equation (19), thereby isolating the magnetic contribution. Initial meshes of 1000, 2000, and 4000 elements yield 1000–1004, 2000–2004, and 4000–4004 effective elements after interface-node insertion and coincident-node merging. Numerical and analytical solutions are compared at the ends, quarter points, and midpoint of each element, using matching one-sided limits at steps. Errors are normalized by the sampled maximum analytical-field magnitude. Across the three meshes, the maximum relative errors are 8.57 × 10^−5^, 2.17 × 10^−5^, and 5.45 × 10^−6^ for the step-field cases and 2.84 × 10^−5^, 7.14 × 10^−6^, and 1.80 × 10^−6^ for the transition cases. Both sets exhibit nearly second-order convergence. The one-dimensional finite-element assembly and the independent shooting/collocation checks were implemented with in-house scripts in MATLAB R2018b; the nonlinear boundary-value calculation uses MATLAB’s bvp4c solver. These comparisons check the discretization and the linearization within the same one-dimensional reduction, not its lateral-flux or few-carrier accuracy.

Figure 2a,b compares the linear and nonlinear potentials and electric fields at zero magnetic increment and at *A* = −0.8. The solutions nearly overlap at the displayed scale. Figure 2c resolves their field difference at *A* = −0.16, −0.3, −0.8, with the largest deviation near the field-region edges. Across the five cases *A* = 0, −0.16, −0.3, −0.5911208, −0.8, the maximum sampled relative field error is 1.79 × 10^−5^, indicating a small effect of drift linearization on the electric-field profiles for these weak-junction parameters. In Figure 2d, dimensionless integration steps of 0.05, 0.025, 0.0125, and 0.00625 yield absolute center-field errors of 9.77 × 10^−6^, 5.86 × 10^−7^, 3.62 × 10^−8^, and 2.26 × 10^−9^, respectively. Halving the step reduces the error by approximately a factor of 16, consistent with fourth-order Runge–Kutta convergence.

### 5.2. Magnetic Amplitude and Junction-Field Regulation

**Figure 3:**
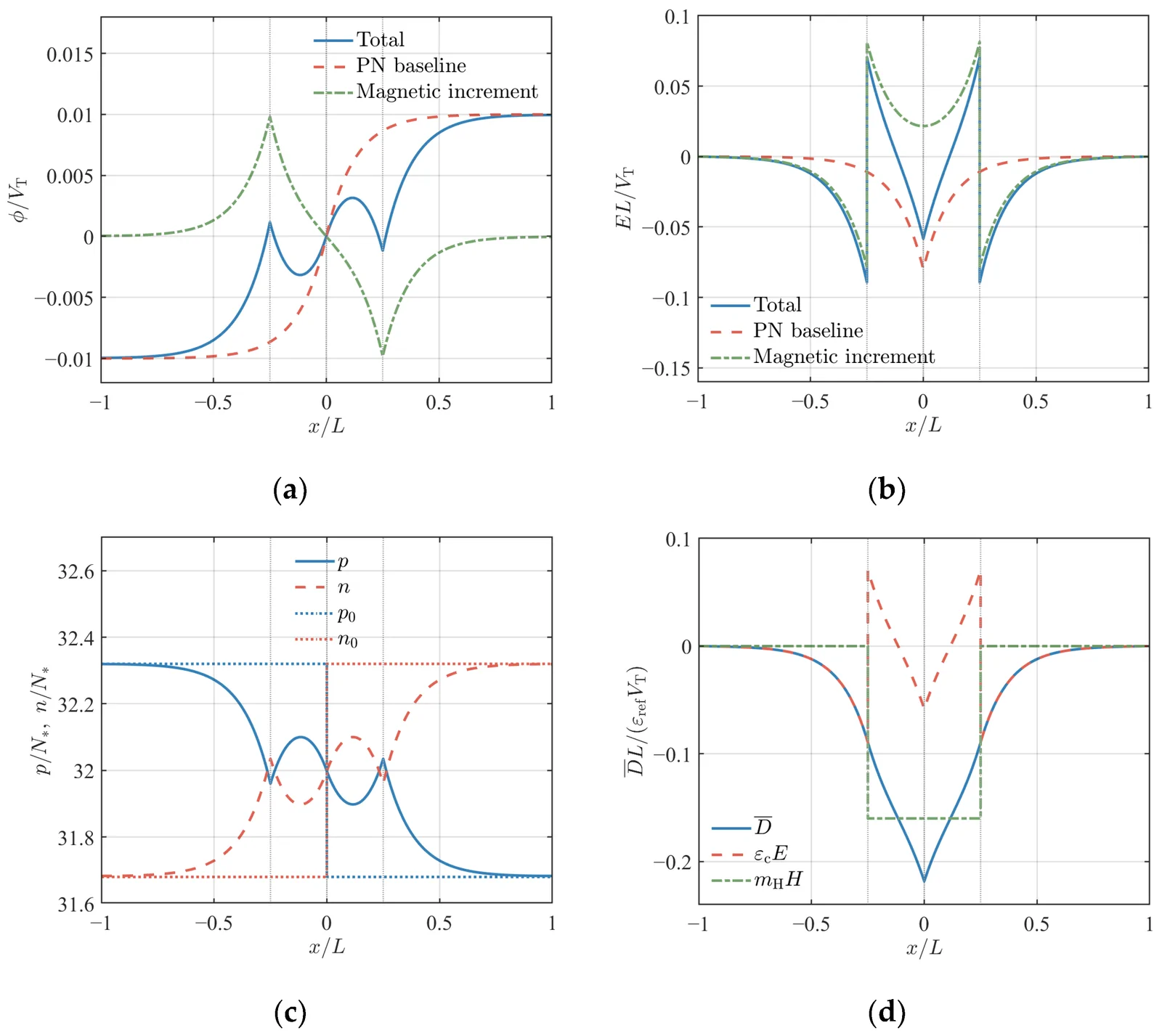
Field distributions for the baseline parameters: (**a**) total potential and its PN-junction and magnetically induced components; (**b**) corresponding electric fields; (**c**) actual hole and electron concentrations and their reference values; and (**d**) electric displacement normalized by the semiconductor area, together with its electric-field and magnetically induced contributions.

Figure 3a shows the superposition of the baseline junction potential and the magnetically induced electric potential. Carrier redistribution near the doping interface establishes the baseline junction field, while opposite-sign potential variations at the magnetic boundaries modify the gradient in the central region. In Figure 3b, the baseline center field ${\hat{E}}_{\mathrm{PN}}(0)=-0.0800$ is partly offset by the magnetically induced component ${\hat{E}}_{H}(0)=0.02165$, giving $\hat{E}(0)=-0.05835$. The magnetically induced electric-field contribution decays from the field-region edges toward the junction, with its center amplitude set by screening over this distance. The electric field jumps at the magnetic boundaries but remains continuous at the PN interface. In Figure 3c, the perturbations compensate the reference-concentration steps, leaving the actual hole and electron concentrations continuous at all three interfaces. Figure 3d shows equal and opposite jumps in the electric-field and magnetically induced contributions to the electric displacement at the field-region edges, preserving continuity of the total electric displacement.

**Figure 4:**
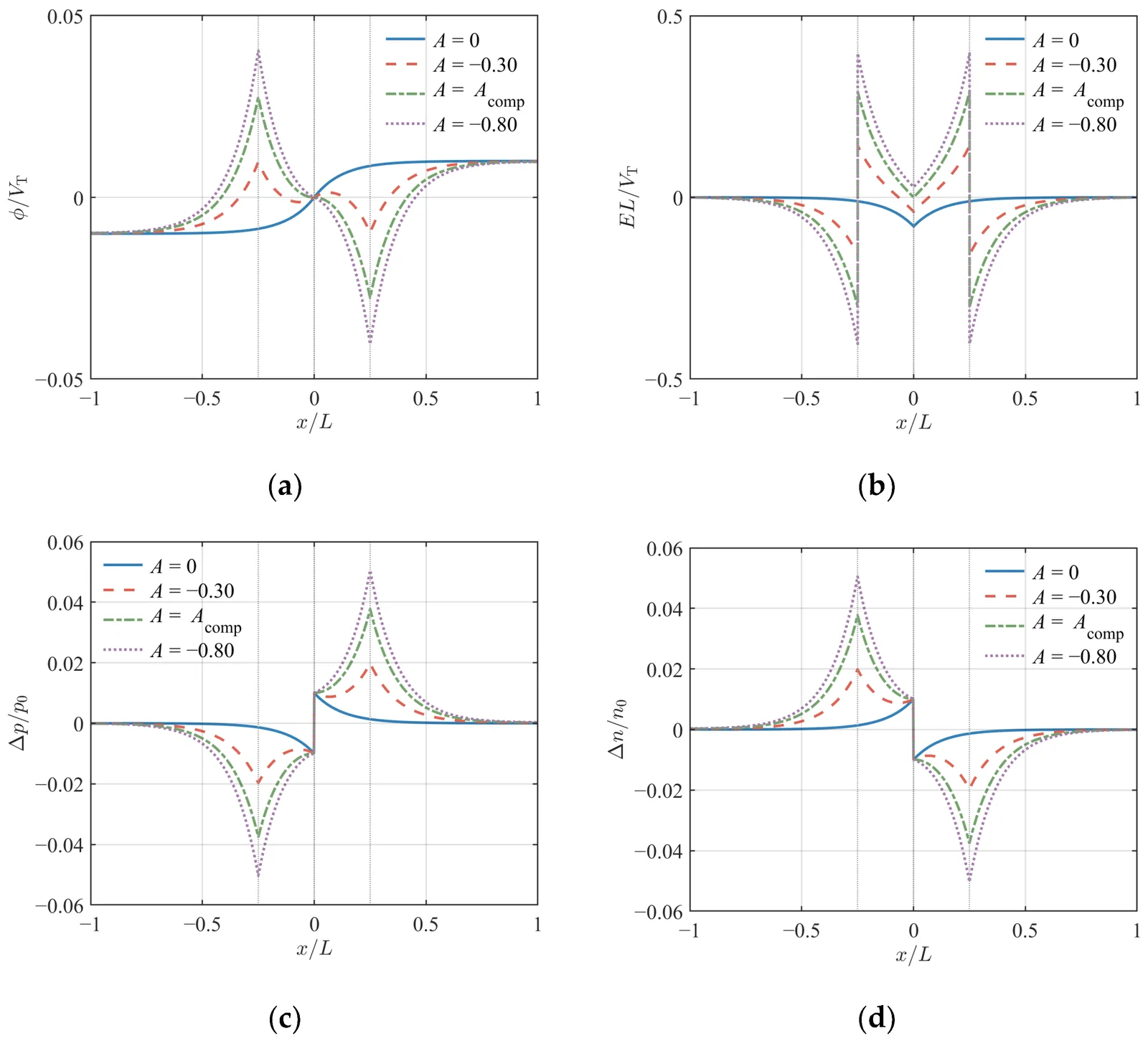
Effects of the signed dimensionless magnetic-field increment *A* on (**a**) potential; (**b**) electric field; (**c**) relative hole perturbation; and (**d**) relative electron perturbation. The four curves correspond to *A* = 0, −0.30, *A*_comp_, and −0.80, where *A*_comp_ ≈ −0.5911 cancels the junction-center electric field. Negative values denote negative magnetic increments about the prescribed bias, not a negative total bias field.

The dimensionless magnetic-increment amplitudes *A* = 0, −0.3, −0.5911, −0.8 in Figure 4 give center fields of −0.0800, −0.03940, 0, 0.02827, respectively; the compensation field is evaluated using the unrounded value in Equation (33). As loading increases in the compensating direction, the center potential gradient in Figure 4a decreases in magnitude and changes sign. The center field in Figure 4b correspondingly undergoes suppression, cancellation, and reversal. Fields near the field-region edges remain appreciable after center compensation because the magnetically induced contribution originates primarily at these boundaries. The hole and electron concentration gradients in Figure 4c,d have opposite directions, with their overall offsets determined by the reference concentrations and population conservation. The maximum relative concentration perturbations are approximately 1.00%, 1.98%, 3.77%, and 5.06% for the four linear solutions; the nonlinear value at the largest magnetic amplitude is approximately 5.17%. Center-field reversal is thus achieved with small carrier redistribution for this parameter set.

**Figure 5:**
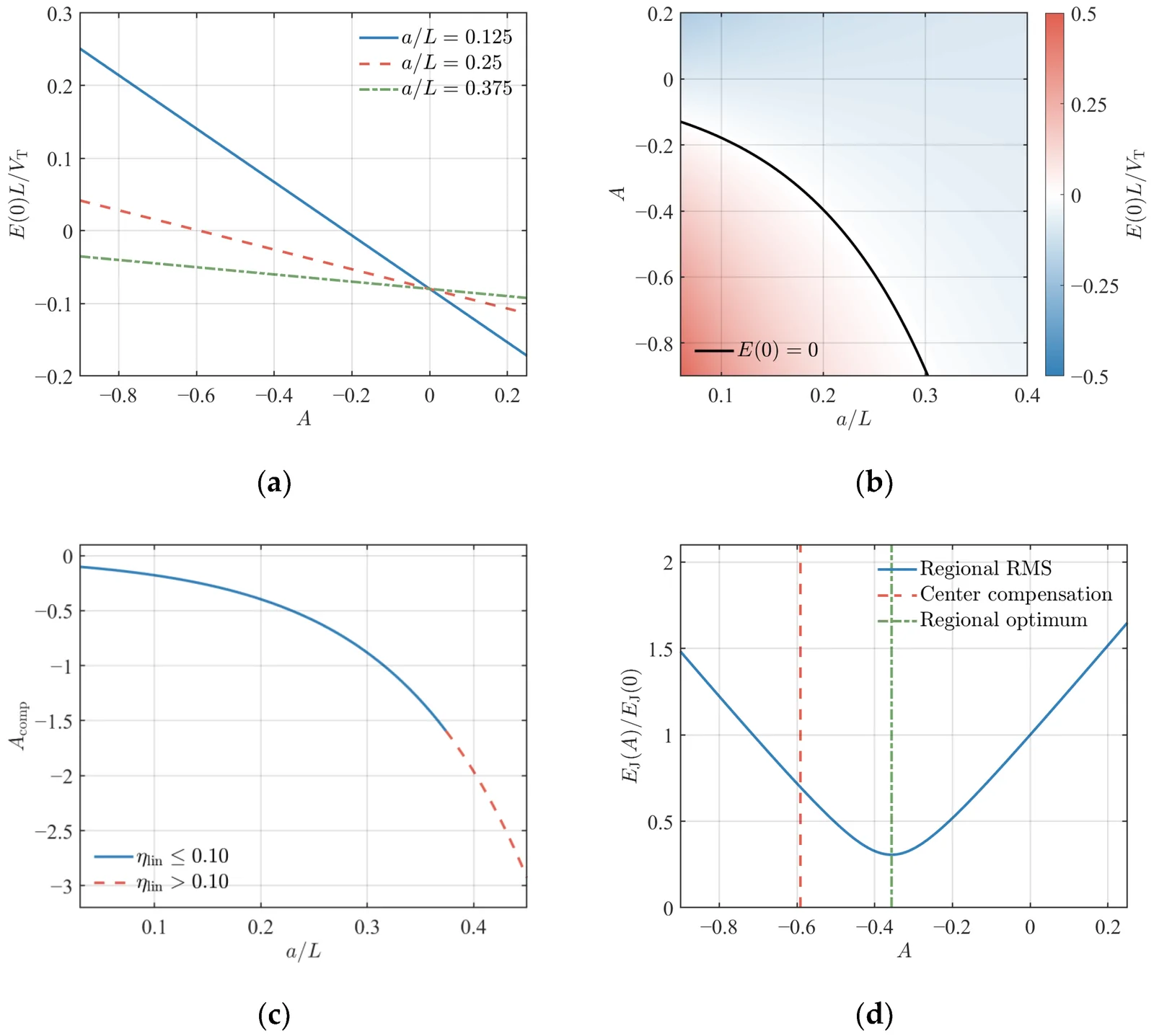
Center-field compensation and suppression over a finite junction region: (**a**) center field versus magnetic amplitude for different field-region widths; (**b**) center-field map and zero-field contour in the amplitude–width plane; (**c**) compensation amplitude, with solid and dashed segments indicating maximum relative carrier perturbations at or below and above 10%, respectively; and (**d**) regional root-mean-square field and the two optimal loading amplitudes.

In Figure 5a, the center electric field varies linearly with magnetic amplitude, but the slope magnitude decreases as the field-excited region widens. Moving the magnetic boundaries away from the junction increases screening attenuation, so a larger magnetic magnitude is required to cancel the same baseline junction field. The black zero-field contour in Figure 5b separates regions that retain and reverse the baseline field direction. It reaches the plotted amplitude limit *A* = −0.9 at *a*/*L* ≈ 0.3025; compensation for wider field-excited regions lies outside the displayed range. Figure 5c combines the compensation curve with the concentration-perturbation condition. The criterion *η*_lin_ ≤ 0.10 separates the solid branch from the dashed linear continuation beyond this threshold. This threshold screens the parameter range, while linearization errors are evaluated through the nonlinear calculations described for Figure 2.

Figure 5d gives the root-mean-square field over the fixed interval l = 0.1*L*. The center-compensating amplitude *A*_comp_ = −0.5911 gives *E*_J_(*A*_comp_)/*E*_J_(0) = 0.6971, whereas regional optimization using Equation (38) gives *A*_RMS_ = −0.3566, *E*_J_(*A*_RMS_)/*E*_J_(0) = 0.3062. The baseline junction field decays away from the center, while the magnetically induced compensating electric field increases toward the field-region edges within the field-excited region. Their opposite spatial trends produce overcompensation on either side of the center when the center field is exactly cancelled. Reducing the magnetic magnitude in the compensating direction weakens these reversed fields, yielding a lower regional root-mean-square field.

### 5.3. Loading Width and Carrier Screening

**Figure 6:**
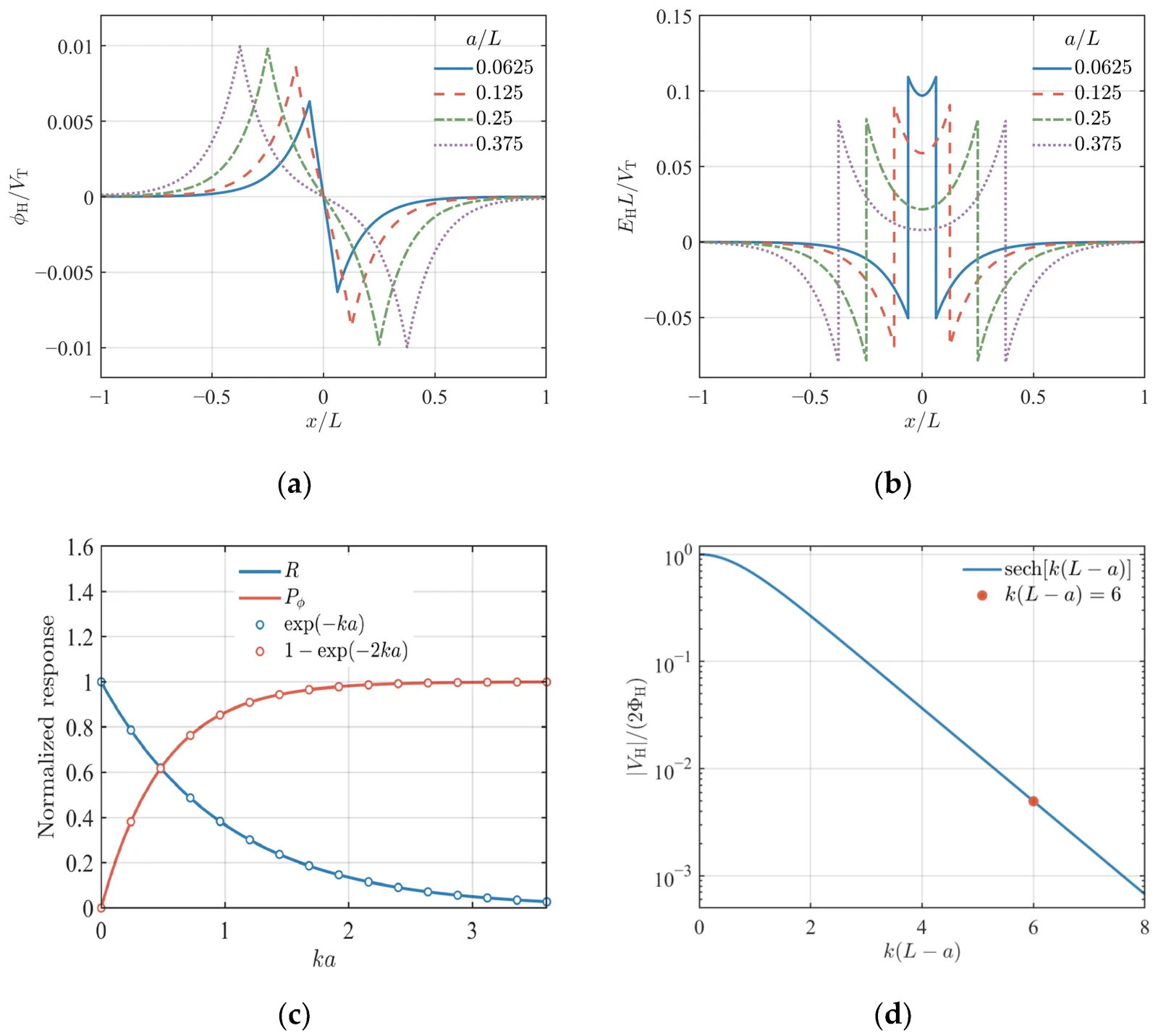
Effects of magnetic-region width: (**a**) magnetically induced electric potential; (**b**) magnetically induced electric-field contribution; (**c**) center-field transfer coefficient and normalized local-potential amplitude; and (**d**) end-to-end potential difference normalized by twice the local-potential amplitude. The field-region edges are *x* = ±*a*; the center and end responses refer to the same fixed fiber length.

In Figure 6a,b, the potential extrema move outward with the magnetic boundaries, while the field reaching the junction center weakens. At *kL* = 8, the values *ka* = 0.5, 1, 2, 3 give center transfer coefficients *R* of approximately 0.6065, 0.3679, 0.1353, and 0.04979 and normalized local potentials *P_ϕ_* of approximately 0.6321, 0.8647, 0.9817, and 0.9976, respectively. Increasing *ka* = 0.5 to *ka* = 2 reduces the center field by approximately 77.7%, even as the boundary potential approaches its plateau in Figure 6c. The asymptotic results from Equation (36) are close to the finite-length values over this range; the final potential estimate is 0.9975, with the difference arising from end corrections. The end-to-end potential difference in Figure 6d is additionally screened between the field-region edges and the fiber ends. Under the baseline condition *k*(*L* − *a*) = 6, |*V_H_*|/(2*Φ_H_*) = sech6 ≈ 0.00496, so the end difference is much smaller than twice the local-potential amplitude. As loading extends toward the ends, this screening weakens, and the finite-length relation in Equation (35) describes the resulting field distribution.

**Figure 7:**
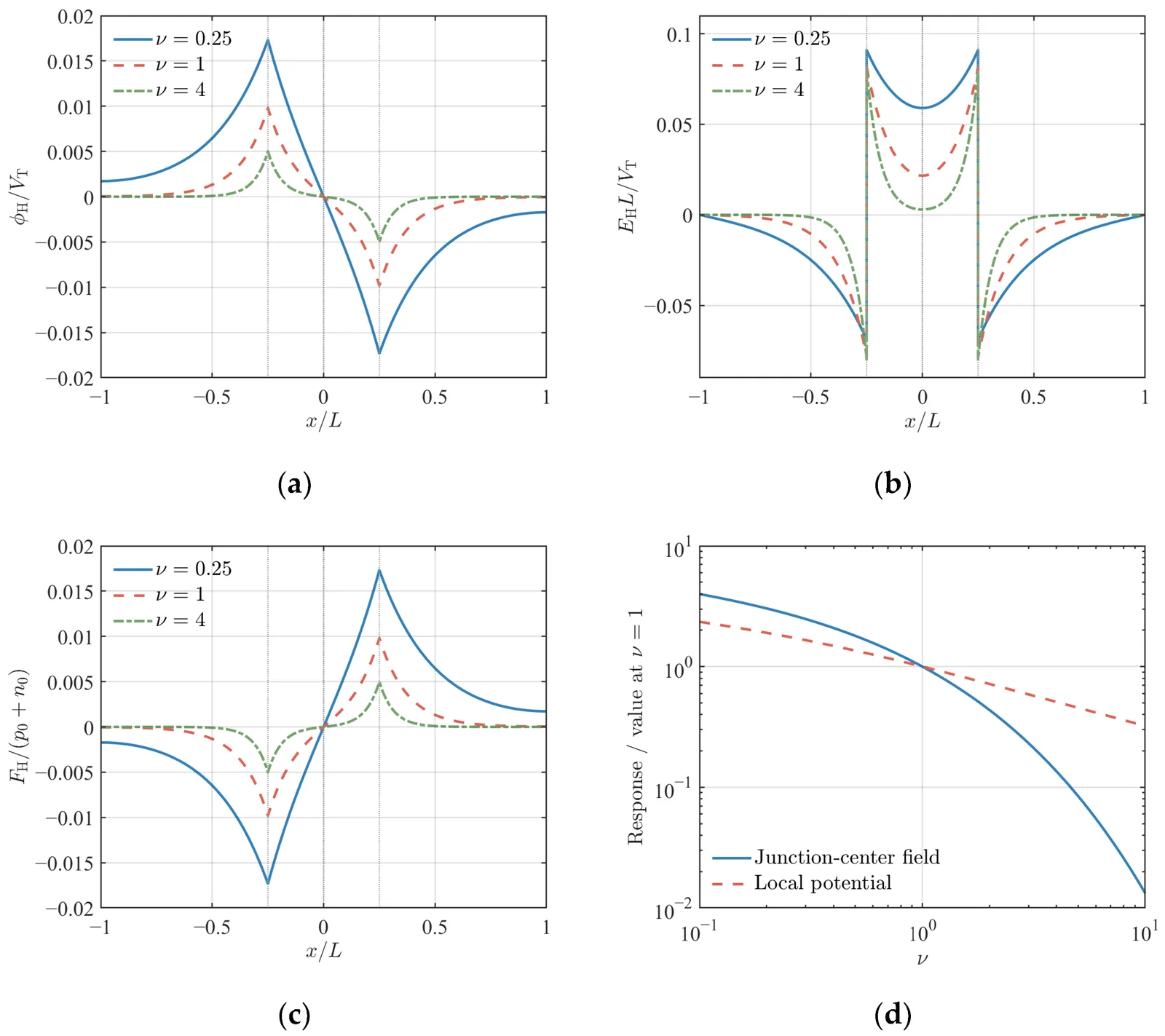
Effects of reference carrier concentration: (**a**) magnetically induced electric potential; (**b**) magnetically induced electric-field contribution; (**c**) net perturbation normalized by the total reference concentration; and (**d**) magnetically induced electric field at the junction center and local potential relative to their baseline values. All reference concentrations are multiplied by *ν*.

Multiplying all reference concentrations by *ν* at fixed concentration ratios, geometry, and applied magnetic field gives $k\propto \sqrt{\nu }$. In Figure 7, *ν* = 0.25, 1, 4 correspond to *kL* = 4, 8, 16, respectively. Increasing concentration shortens the screening length and confines the potential and field in Figure 7a,b more closely to the field-region edges. The center fields are ${\hat{E}}_{H}(0)=0.05899,\;0.02165,\;0.002931$, and the local-potential amplitudes are *Φ_H_*/*V*_T_ = 0.01733, 0.009817, 0.004998. From the lowest to the highest concentration, the magnetically induced electric field at the junction center decreases by approximately 95.0%, compared with 71.2% for the local potential. The center response decays faster because it is also screened over the boundary-to-junction distance. Figure 7c normalizes the net concentration perturbation by the total reference concentration of each case and therefore describes its change relative to the carrier background. Figure 7d compares the relative attenuation of the field and potential. Stronger screening at higher concentrations makes center-field regulation more sensitive to the proximity of the field-region edges to the junction.

**Figure 8:**
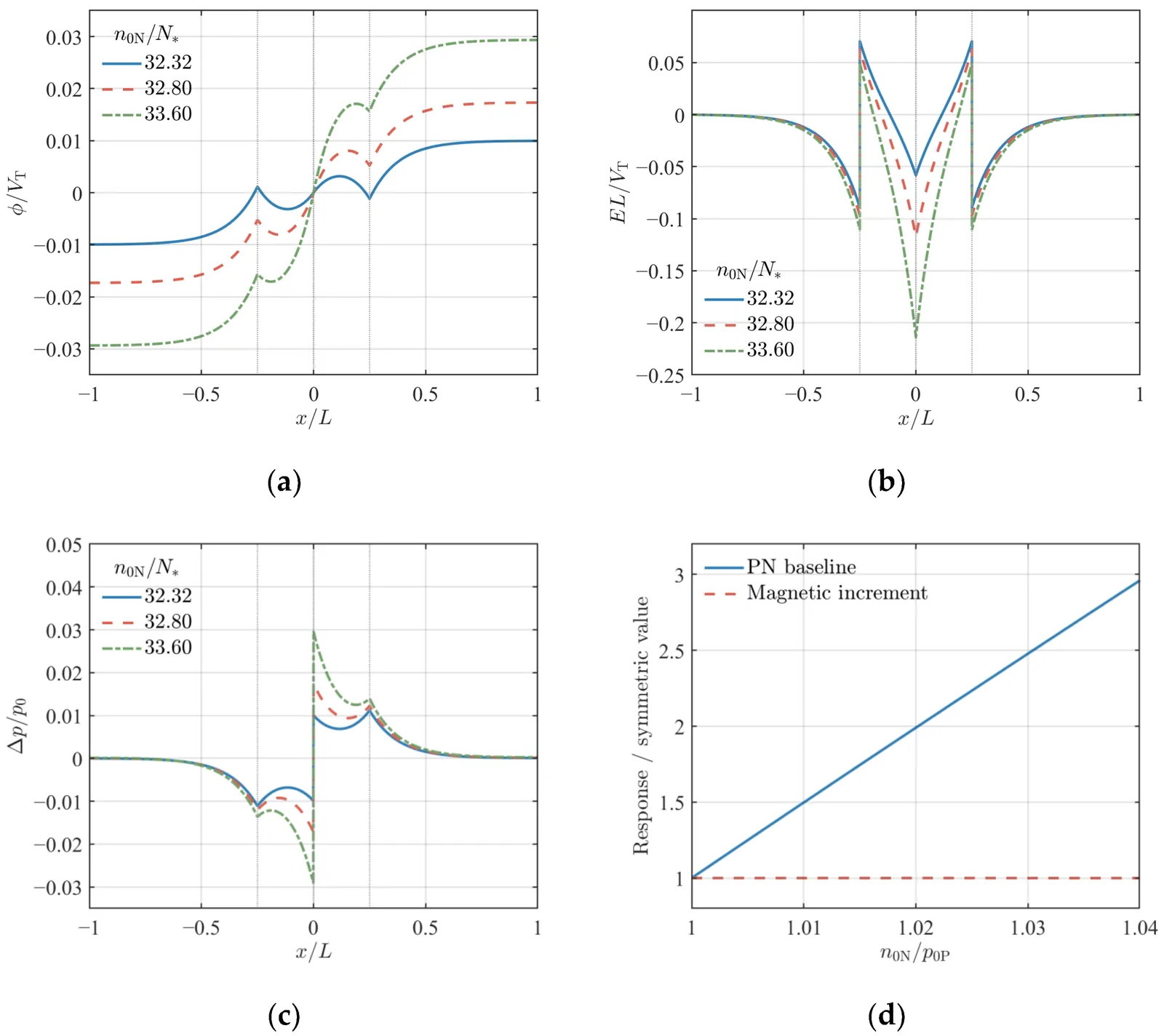
Effects of weak doping asymmetry: (**a**) total potential; (**b**) total electric field; (**c**) relative hole perturbation; and (**d**) baseline and magnetically induced electric-field components at the junction center, each normalized by its value in the symmetric case.

Figure 8 fixes the P-region reference concentrations and increases the N-region electron reference concentration subject to *p*_0N_*n*_0N_ = *p*_0P_*n*_0P_. The values *n*_0N_/N_*_ = 32.32, 32.8, 33.6 correspond to *p*_0N_/N_*_ = 31.68, 31.2164, 30.4731, respectively. In Figure 8a–c, the baseline center-field magnitude increases from 0.0800 to 0.2354, whereas the magnetically induced electric-field increment changes only from 0.021654 to 0.021641, or approximately 0.057%. According to Equation (32), the baseline field depends directly on the net concentration mismatch, while the magnetically induced contribution depends on screening through the total reference concentrations. For this parameter set, the mismatch increases substantially, but the totals change little, strengthening the junction field while leaving the magnetically induced component nearly unchanged. Figure 8d uses *n*_0N_/*p*_0P_ as the horizontal coordinate and normalizes each component by its symmetric-case value, displaying this difference directly.

### 5.4. Layer Thickness and Magnetic-Boundary Profile

**Figure 9:**
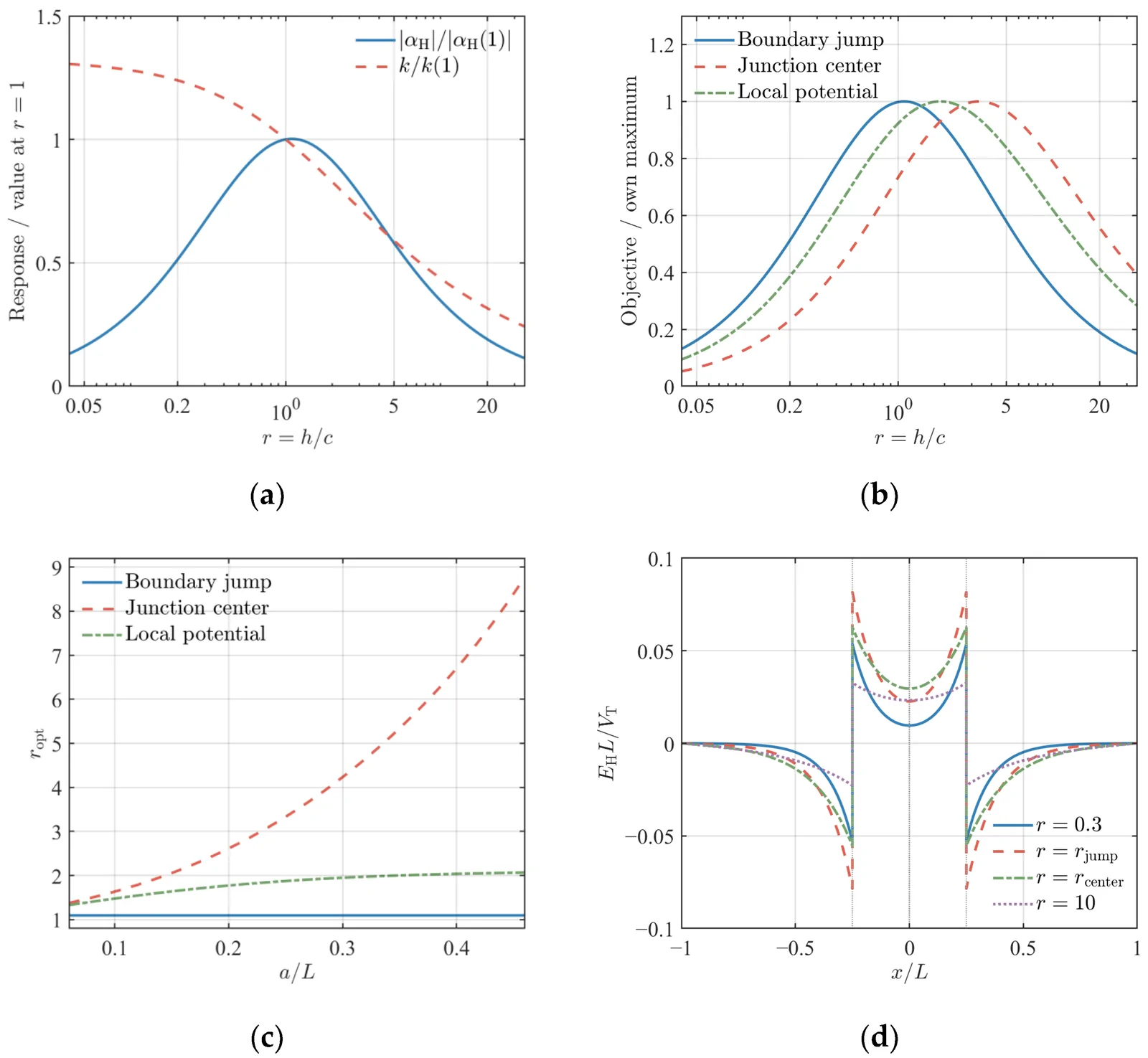
Effects of thickness ratio: (**a**) magnetoelectric conversion coefficient and screening parameter; (**b**) three objective functions normalized by their respective maxima; (**c**) optimal thickness ratios versus magnetic-region width; and (**d**) magnetically induced electric-field profiles for different thickness ratios.

Figure 9 uses a separate dimensionless material set, $c_{m}/c_{s}=1,\varepsilon_{m}/\varepsilon_{s}=1,e_{s}^{2}/c_{s}\varepsilon_{s}=0.2$, to examine layer-thickness effects. Physical reference concentrations, magnetic field, fiber length, width, and total thickness are held fixed. The reference structure at *r* = 1 has *kL* = 8, and Equation (39) determines the effective dielectric coefficient and screening parameter at other thickness ratios. This material set is specified independently of the preceding ZnO/CoFe_2_O_4_ case.

In Figure 9a, the magnetoelectric conversion coefficient first increases and then decreases with piezomagnetic-layer fraction, whereas the screening parameter decreases as the effective permittivity increases. At *a*/*L* = 0.25, Equation (40) and continuous one-dimensional optimization give *r*_jump_ = 1.095, *r_Φ_* = 1.876, and *r_E_* = 3.333 for the maxima of the boundary field jump, local potential, and junction-center field in Figure 9b, respectively. The center-field optimum contains a larger piezomagnetic fraction than the boundary-jump optimum: although local conversion has begun to decline, the longer screening length still increases the field reaching the center. In Figure 9c, the center-field optimum moves to larger thickness ratios as the field-excited region widens. The boundary-jump optimum is independent of field-region width, and the local-potential optimum lies between the two. The profiles in Figure 9d correspondingly show center-field enhancement accompanied by a lower boundary peak, reflecting the combined effects of magnetic driving strength and screening length on the spatial field distribution.

**Figure 10:**
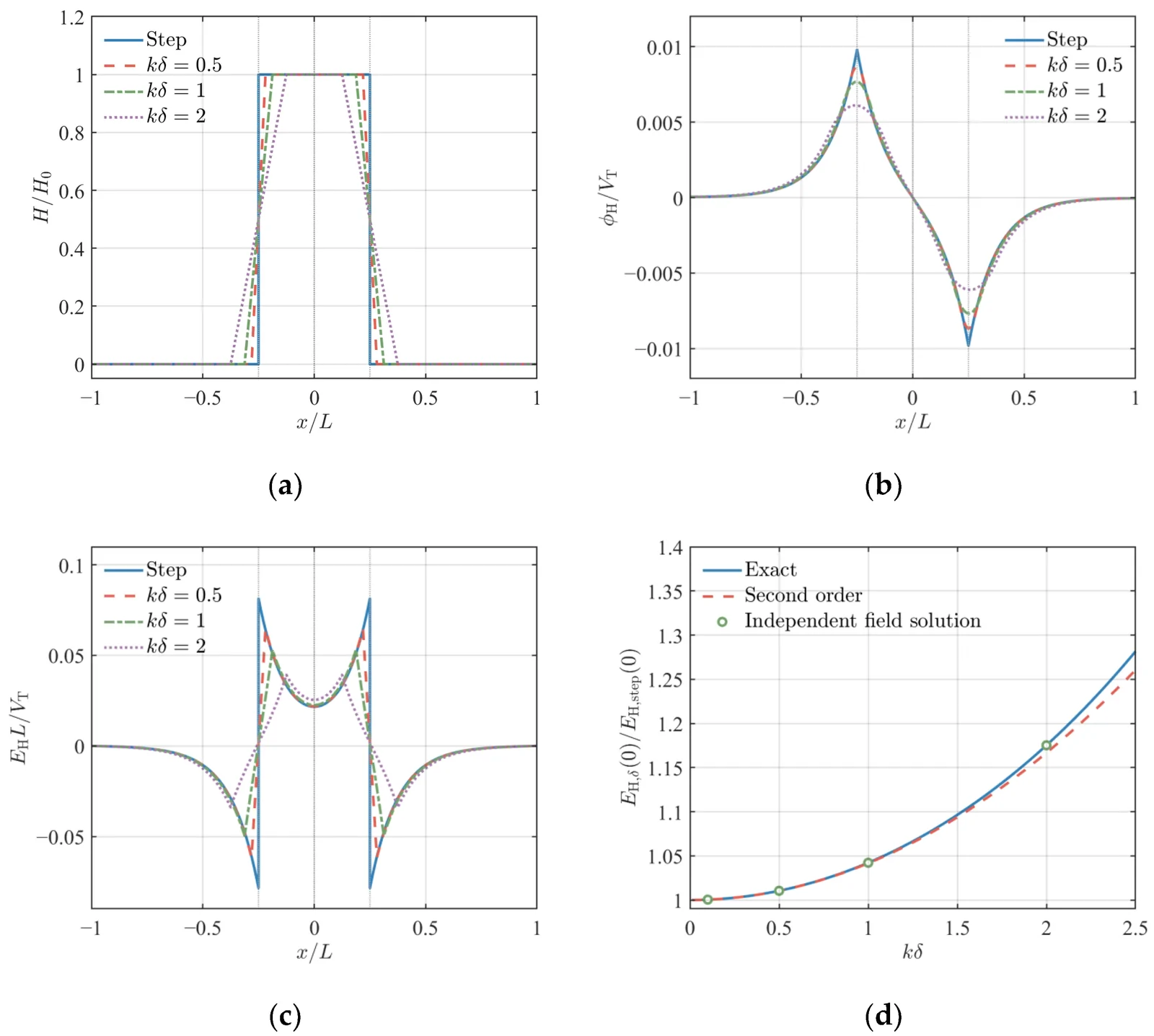
Effects of finite magnetic transition width: (**a**) magnetic-field profiles; (**b**) magnetically induced electric potential; (**c**) magnetically induced electric-field contribution; and (**d**) exact center-field correction, second-order approximation, and independent piecewise calculations.

Figure 10a compares the step field with trapezoidal profiles for *kδ* = 0.5, 1, 2, all having the same peak amplitude and axial integral. A finite transition distributes the driving source previously concentrated at the boundary. The potential in Figure 10b remains continuous. In Figure 10c, smoothing removes the electric-field jump, lowers the peak, and broadens the response. At *kδ* = 2, the maximum magnetically induced electric-field contribution decreases from 0.08147 to 0.03927, or approximately 51.8%, and the local magnetically induced electric potential decreases from 0.009817 to 0.006112, or approximately 37.7%. Meanwhile, the center field increases from 0.02165 to 0.02545, or approximately 17.5%. The part of the transition closer to the junction experiences a shorter screening distance; its increased contribution exceeds the reduction from the outer part, in agreement with Equation (42). The piecewise numerical results in Figure 10d coincide with the exact relation, while the second-order approximation increasingly departs from it as transition width grows. For this trapezoidal profile, the step approximation overestimates the boundary peak and underestimates the center field when the transition width is comparable to the screening length.

These results describe axial electrostatic responses in a linear piezomagnetic operating regime with carrier-blocking boundaries. The finite-element and nonlinear calculations share the same one-dimensional electromechanical reduction, assessing spatial discretization and drift linearization, respectively; interlayer shear, transverse fields, and demagnetization are not included.

### 5.5. Potential-Difference Signal, Readout Position, and the Role of the PN Junction

**Figure 11:**
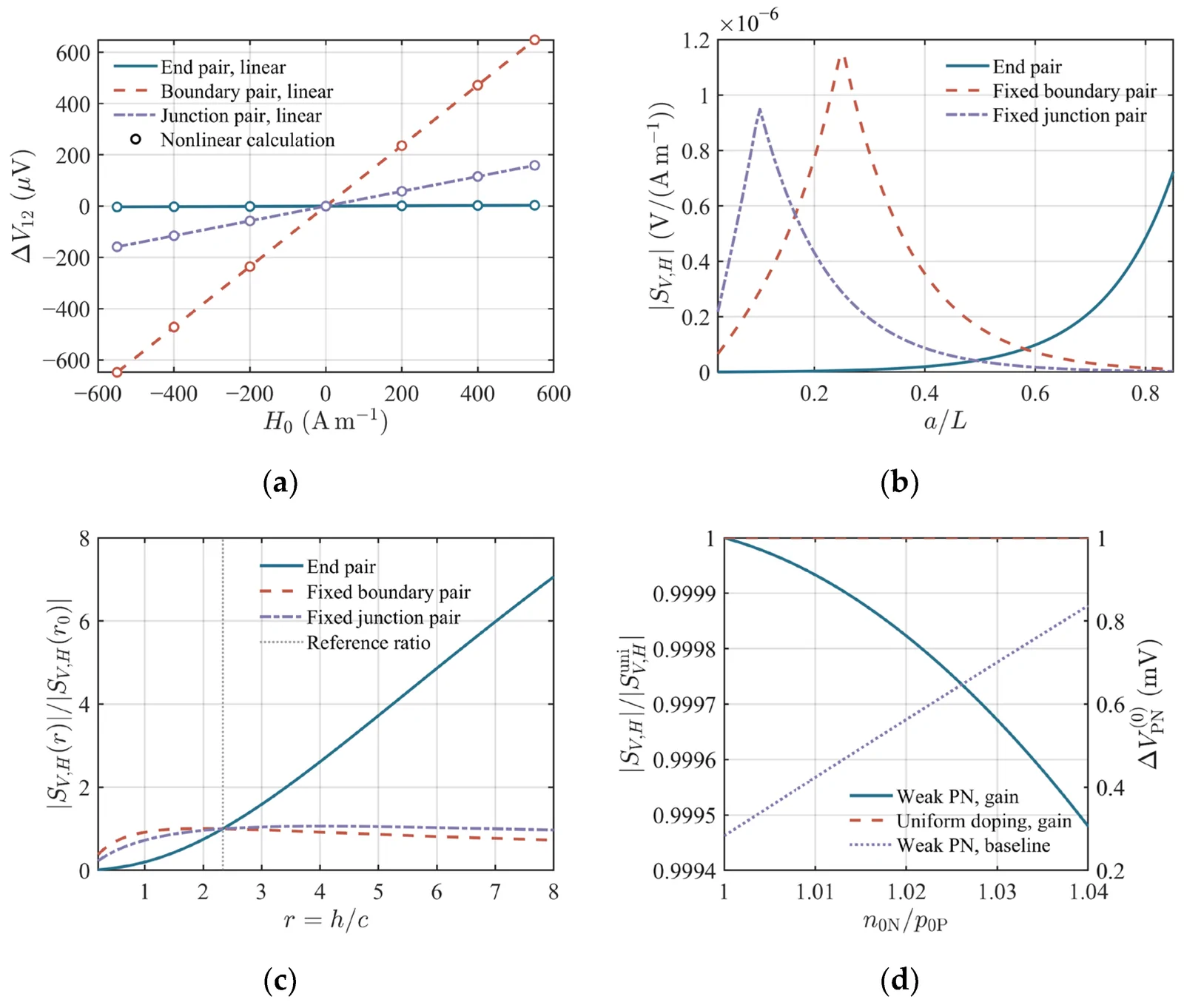
Magnetically induced internal potential-difference response: (**a**) fixed-pair response versus magnetic-field increment, with nonlinear drift–diffusion markers; (**b**) response coefficient versus loading half-width for fixed observation pairs; (**c**) normalized response coefficient versus layer-thickness ratio using the stated ZnO/CoFe_2_O_4_ constants; and (**d**) a uniformly doped control compared with symmetric and weakly asymmetric PN-junction reference states. The response is not a contact-corrected sensor terminal voltage. The three fixed symmetric readout pairs are the fiber ends (±4 μm), the reference field-region edges (±1 μm), and the junction vicinity (±0.4 μm); these locations do not move during the width scan.

Figure 11a places the end pair, a pair fixed at the reference field-region edges, and a junction-centered pair of half-spacing 0.4 μm on the same magnetic scale. At an increment of −427.65 A/m, their internal potential differences are −2.50, −504, and −123 μV, with respective response coefficients of 5.845 × 10^−9^, 1.179 × 10^−6^, and 2.887 × 10^−7^ V/(A m^−1^). All three pairs experience the same prescribed internal magnetic-field increment; their different signals arise from their positions relative to the field-region edges and the carrier-screened junction. The magnetic source produces its largest potential variation near the field-region edges; the junction pair samples the field after part of that response has been screened, while the end pair lies farther away. The edge and junction pairs therefore produce differences about 200 and 50 times the end-to-end value. Nonlinear drift–diffusion markers provide a separate check of carrier linearization over the plotted increments; the linear analytical curves alone cannot establish a device operating range. Because the reported quantity subtracts the zero-increment reference, it is independent of the arbitrary additive constant in electric potential and isolates the field-induced part of the PN-junction response. The marked reduction at the fiber ends is therefore a transfer-and-screening effect, not evidence that the local magnetic-to-polarization conversion vanishes.

Figure 11b tests whether extending the field-excited segment improves a fixed readout. When an edge lies between the junction and an interior observation point, moving it toward that point shortens the screened transfer path and increases the response. After the edge passes the point, further widening separates the source from the fixed pair and reduces the response. Equations (45)–(47) therefore predict a peak when the edge reaches an interior readout point. The end pair has no corresponding interior crossover. Figure 6d shows the related attenuation at the fiber ends: for the reference geometry, the end-to-end difference is about 1% of the peak local magnetically induced potential. This compares electrical potentials, not magnetic-field amplitudes. A finite-width field transition would smooth the sharp peak predicted for an ideal step profile.

The layer-ratio scan in Figure 11c changes the strength of the magnetic source and the degree of screening simultaneously. Increasing the piezomagnetic fraction first strengthens the transferred strain and its induced polarization, but it also changes the section stiffness and effective permittivity, and hence the screening parameter. A fixed pair samples the product of this source factor and a position-dependent spatial transfer factor; optimizing either factor separately does not optimize their product. With the stated material constants and fixed total thickness, the normalized gain peaks near ratios of 2.0 and 4.0 for the reference-boundary and junction pairs, while the end-pair gain still rises over the displayed interval. The differing peak positions reflect their differing screening paths. Figure 9 uses an independent dimensionless material group, so its thickness optimum is a general parametric result, not a numerical prediction for the material combination used in Figure 11c. No unrestricted device optimum follows from the finite interval shown. In mechanistic terms, changing the layer ratio modifies both the effective magnetically induced polarization source and the characteristic decay of its carrier-screened field. The reported peak is consequently a property of the specified material set, total thickness, and readout pair, rather than a transferable optimum for other composites [36,37].

Figure 11d isolates the role of the PN interface. The uniformly doped control retains the same geometry and total reference carrier concentration but has no doping step. When its screening parameter equals that of the symmetric weak junction on both sides, the two structures have the same magnetically induced potential-difference response. Their zero-increment reference potentials differ because only the PN specimen contains a doping interface. Raising the N-side electron reference concentration by 4%, while retaining the specified concentration product, changes the zero-increment difference across the fixed junction-centered readout pair from 0.283 to 0.837 mV; the magnetic response coefficient changes by only 0.052%. The weak junction therefore provides a controllable reference field in this comparison, not an inherent increase in magnetic conversion gain. Larger doping contrasts require a separate nonlinear assessment [38,39].

The fixed-pair comparisons describe the internal small-signal response under the prescribed magnetic and electrical conditions. The following subsection examines the carrier-count interpretation, geometric similarity, and sensitivity to electrical termination.

### 5.6. Continuum Scaling and Electrical Boundary Sensitivity

The reference populations require a separate assessment from the agreement between analytical and numerical solutions. The stated semiconductor volume of 2.4 × 10^−20^ m^3^ and total reference density of 1.813 × 10^20^ m^−3^ correspond to about 4.35 mobile carriers in the full fiber and 0.272 carriers in a semiconductor segment one screening length long. The transverse dimension is 0.0125 of the full length but 0.20 of the screening length, so global slenderness alone does not quantify the accuracy near screened field variations. Smooth deterministic densities are not quantitatively justified for a single specimen at this mapping. The weak concentration imbalance corresponds to a net dopant-charge equivalent of about 0.0218 elementary charges per half-fiber; this is not the total number of impurity atoms, which could be larger in a compensated material. The physical-unit curves are predictions of the specified continuum equations, with a realizable carrier state and representative averaging volume still to be established.

The dimensionless solution nevertheless has an explicit geometric similarity. With material coefficients and temperature fixed, the simultaneous transformations (*L*, *a*, *b*, *c*, *h*) → *s*(*L*, *a*, *b*, *c*, *h*), (*p*_0*r*_, *n*_0*r*_) → (*p*_0*r*_, *n*_0*r*_)/*s*^2^, and *H*_0_ → *H*_0_/*s* preserve the dimensionless screening parameters, magnetic amplitude, reference concentration ratios, and field-region geometry. The dimensionless potential profiles remain unchanged, while the carrier count in a geometrically similar volume increases in proportion to the length scale factor. For example, a factor of 1000 gives about 4350 carriers in the full fiber and 272 per screening-length segment. The transverse-to-screening-length ratio remains 0.20 under this transformation. This is a mathematical scaling relation, not a demonstrated material design: the near-equal carrier populations, constitutive coefficients, and preparation timescale still require independent physical justification.

The end electric-displacement assumption can also be tested within the one-dimensional equations. For equal screening, let *δD*(±*L*) = *D*_b_ at both ends relative to the same zero-increment reference. Equal values at both ends preserve the integrated free charge in the absence of lateral flux. The perturbation is the change between the loaded and reference states; a field-independent offset identical in both states cancels from the linear magnetic difference. Subtracting the original solution gives a homogeneous screening problem with the exact correction *δE*(*x*) = (*D*_b_/*ε*_c_)((cosh(*kx*))/(cosh(*kL*))). The corresponding change in potential difference across (−*ξ*,*ξ*) is *δV*(*ξ*) = −((2*D*_b_)/(*ε*_c_*k*))((sinh(*kξ*))/(cosh(*kL*))). These expressions satisfy the incremental Gauss law, both end conditions, and continuity at the internal interfaces.

For a nonzero magnetic increment, define *η*_b_ = |*D*_b_/(*m_H_H*_0_)| and normalize the boundary-induced change by the unperturbed magnetically induced readout. The end pair gives *r*_end_ = *η*_b_(sinh(*kL*))/(sinh(*ka*)); a fixed inner pair satisfying 0 < *ξ* ≤ *a* gives *r*_inner_ = *η*_b_sech(*k*(*L* − *a*)). At *kL* = 8 and *a*/*L* = 0.25, these multipliers are about 411 and 0.00496, respectively. A common incremental end electric displacement equal to 0.1% of the magnetic source therefore changes the weak end-to-end response by about 41%, but the inner-pair response by only about 0.00050%. This is a conditional boundary-sensitivity calculation, not an estimate of an actual contact field. It shows that end readout is particularly sensitive to departures from the assumed electrical termination.

The calculation above addresses only a charge-compatible end perturbation. Lateral displacement flux requires the transverse fields and surrounding dielectric domain, which the present model does not determine; a numerical percentage cannot be inferred from slenderness alone. Similarly, the selected CoFe_2_O_4_ constants do not determine an external magnetic bias, hysteresis, or demagnetizing correction. The resulting readout coefficients describe the internal small-signal response of the stated continuum model. Device voltage, usable field range, noise, and detection limit are outside the quantities computed here.

### 5.7. Self-Consistent Carrier Statistics and Readout Robustness

In the equilibrium-state comparison, the temperature is 298 K, and the band gap is 3.37 eV. The effective densities of states at 300 K are 2.2 × 10^24^ and 1.8 × 10^24^ m^−3^ for the conduction and valence bands, respectively [46]; both are scaled with temperature to the three-halves power. These literature simulation inputs give an intrinsic concentration of 6.277 × 10^−5^ m^−3^ at 298 K. The magnitude of the fully ionized donor or acceptor concentration is 1.0 × 10^21^ m^−3^. The original geometry and reduced electromechanical coefficients are retained only for a controlled comparison. The magnetic field is centered, its nominal half-width is 1.0 μm, and each edge has a linear transition width of 0.24 μm. The three fixed pairs are (−0.4, 0.4), (−1.0, 1.0), and (−4.0, 4.0) μm.

**Figure 12:**
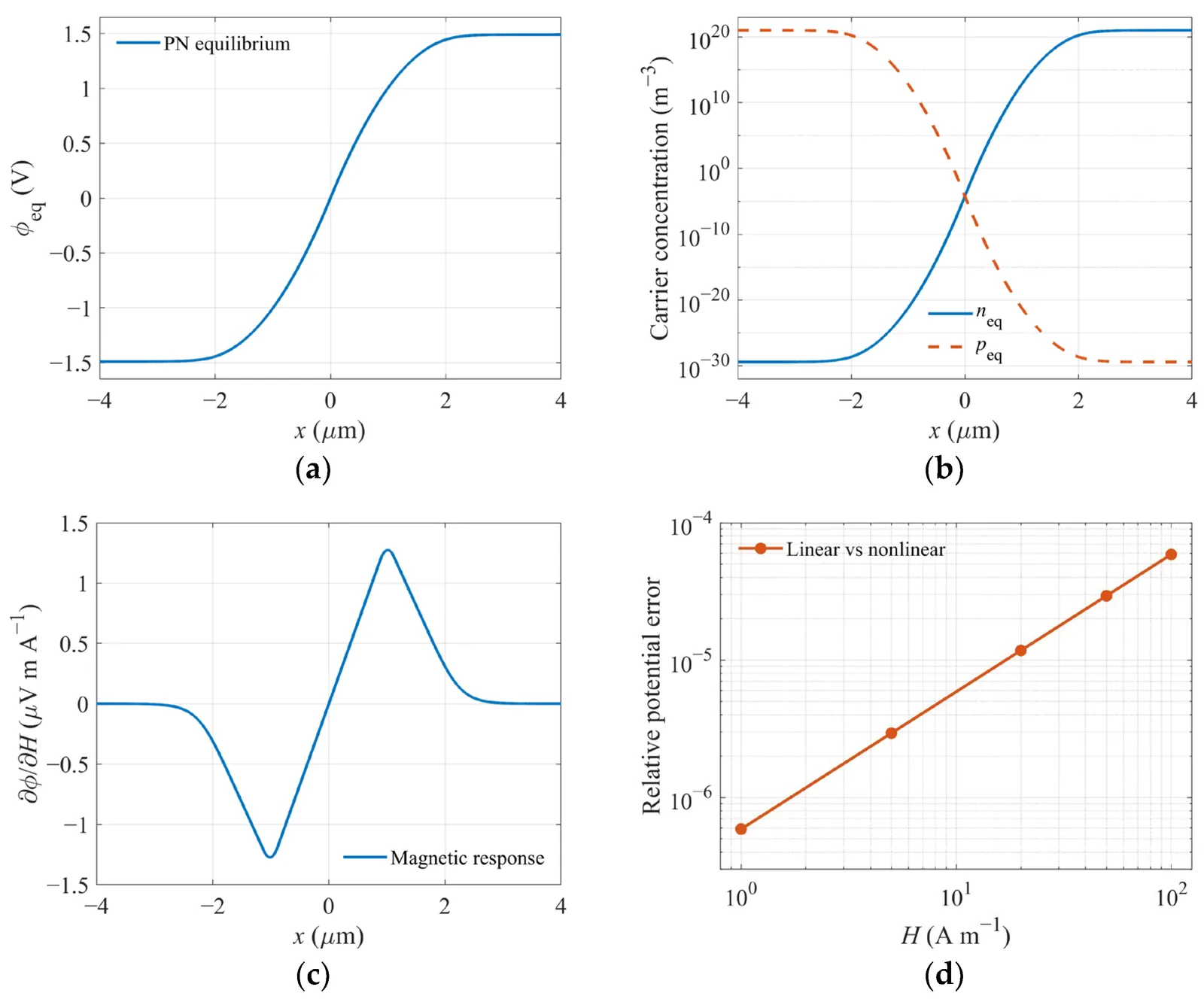
Additional equilibrium-state comparison with the original geometry: (**a**) equilibrium potential; (**b**) equilibrium electron and hole concentrations; (**c**) magnetic potential derivative with respect to the internal field increment; (**d**) relative maximum difference between the tangent response and the finite-amplitude equilibrium potential difference. The calculation permits equilibrium pair generation and recombination and does not separately constrain total electron and hole numbers. It is not the original weak-junction reference state.

Figure 12 distinguishes the built-in equilibrium state from its magnetic increment. The equilibrium potential difference is 2.9804 V, and the electron and hole profiles satisfy the mass-action relation throughout the junction. The depletion region has weaker screening than the quasi-neutral ends, so the incremental potential is concentrated internally. The magnetic potential-difference response coefficients of the junction, interior, and end pairs are 1.076, 2.547, and 0.0001791 μV m A^−1^, respectively. The internal-to-end contrast is therefore retained in this equilibrium model; however, its magnitude is not the approximately 200-fold contrast of the original weak-junction example. The very small end response also makes a large ratio alone unsuitable as a device-performance metric.

The equilibrium equation was solved with a damped Newton method and a mass-lumped, linear finite-element discretization on the half-domain. Exact element integrals were used for the trapezoidal magnetic source. An independent MATLAB R2018b bvp4c calculation gives maximum relative potential differences of 1.17 × 10^−8^ for the reference state and 4.96 × 10^−8^ for its tangent response. Increasing the half-domain mesh from 2000 to 4000 elements changes the three readout coefficients by at most 1.71 × 10^−5^ relative to the finer result. The finite-amplitude equilibrium difference was solved directly using exponential increments to avoid subtraction errors. Between 1 and 100 A/m, the relative maximum difference from the tangent potential increases from 5.88 × 10^−7^ to 5.88 × 10^−5^, with both potentials referenced to the junction center.

**Figure 13:**
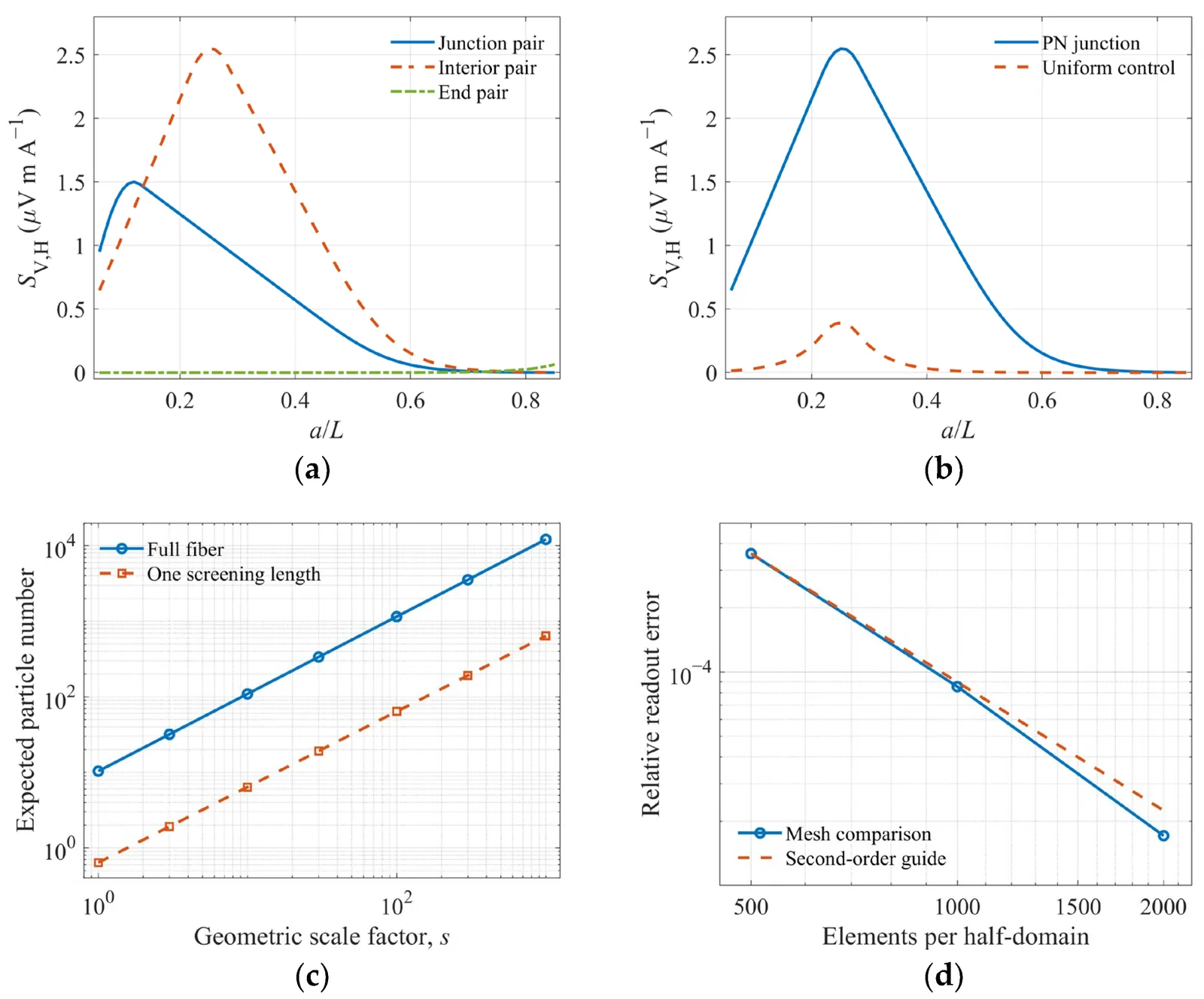
Readout geometry, screening control, and statistical scaling in the equilibrium comparison: (**a**) fixed-pair response coefficients versus normalized magnetic half-width; (**b**) interior-pair response compared with a uniform quasi-neutral control; (**c**) expected mobile-carrier counts under geometry-density scaling; (**d**) readout-coefficient convergence relative to 4000 half-domain elements. In (**a**,**b**), observation points remain fixed as the field width changes. The uniform control matches the quasi-neutral carrier sum, not the local PN-junction screening profile.

Figure 13a varies the magnetic half-width while keeping all observation points fixed. The two internal pairs exhibit distinct response maxima because source position and the depleted region jointly control transmission. Figure 13b compares the interior-pair response with a uniform control having the same quasi-neutral carrier sum and identical material, geometry, and source profile. This control does not match the local screening distribution inside the PN junction. Its smaller response demonstrates the effect of depletion-induced screening variation, rather than an increase in the intrinsic magnetoelectric coupling coefficient. It therefore does not contradict the equal-screening result in Figure 11d.

The statistical limitation remains important. At the original dimensions, the equilibrium comparison contains only about 10.36 mobile carriers and 24 ionized dopants; one quasi-neutral screening-length segment contains about 0.639 mobile carriers. Correcting the carrier statistics alone therefore does not justify a deterministic description of a single nanofiber. Figure 13c scales every geometric dimension by a factor *s* and divides the dopant concentration by *s*^2^ while keeping temperature and intrinsic concentration fixed. At *s* = 100, the fiber length is 0.8 mm, the ZnO thickness is 3 μm, the width is 10 μm, and each magnetic layer is 3.5 μm thick. The model then contains about 1151 mobile carriers and 2400 ionized dopants, with about 63.9 carriers per quasi-neutral screening length. The width-to-screening-length ratio remains 0.470. This scaling increases population counts but does not improve that transverse ratio or quantify lateral electric flux.

Unlike the similarity transformation for prescribed carrier populations, this equilibrium scaling is not an exact invariance: the intrinsic concentration cannot be rescaled independently at fixed temperature. The built-in potential changes from 2.9804 to 2.5074 V at *s* = 100. These equilibrium-state results support the spatial screening mechanism under an alternative, internally consistent carrier closure, but do not validate the original nanoscale specimen or establish a universal operating range. A surrounding-medium field calculation and bias-specific magnetic data are still required for quantitative device predictions.

## 6. Conclusions

We obtained a finite-length solution for the potential difference induced by a local magnetic-field increment in a PN-junction sandwich fiber. The analysis separates the zero-increment junction reference state from the field-induced increment and compares fixed readout pairs, a uniformly doped control, and a weakly asymmetric junction. The following findings apply to the defined continuum reference state; they do not by themselves establish a practicable nanoscale ZnO PN junction.

(1) Carrier screening makes readout location decisive. In the representative weak-junction case, the end-to-end potential difference is about 1% of the peak local magnetically induced potential. Fixed pairs at the field-region edges and near the junction give differences about 200 and 50 times the end-to-end value. A large local potential does not, by itself, imply a large end signal.
(2) Under equal screening, an interior pair held fixed while the step-field region widens has maximal response when a field-region edge reaches the observation points. Widening beyond them increases the screening distance and reduces that pair’s response. The end-to-end difference instead increases across the admissible width range. Thus, the preferred field-region width depends on the readout positions.
(3) A uniformly doped control and the symmetric weak PN junction have identical field-induced increments when their screening parameters are equal, although their zero-increment reference potentials differ. A 4% change in the N-side electron reference concentration raises the junction-centered reference difference from 0.283 to 0.837 mV while changing the magnetic gain by only 0.052% in this weak-asymmetry example. The PN junction mainly tunes the reference field in the parameter range examined.
(4) Layer thickness changes both the magnetically induced polarization source and the carrier-screening length, so its best ratio depends on the observation pair. Smoothing the field-region edge lowers the sharp local field peak but can increase the field increment reaching the junction. These trends depend on the stated geometry and bias-linearized material parameters.

The theory identifies how source location, screening, and structural coupling determine internal small-signal potential differences. The physical-unit example remains an illustrative continuum mapping because of its few-carrier population and uncalibrated magnetic operating state. The boundary-sensitivity analysis further shows that a weak end-to-end signal can change substantially under a small change in end electric displacement, whereas interior readout is less affected in the equal-screening case. Quantitative device predictions require a physically realizable carrier state, bias-dependent magnetic coefficients, and electrical boundary conditions appropriate to the intended contacts and surrounding medium.

## Data Availability

The original contributions presented in this study are included in the article. Further inquiries can be directed to the corresponding authors.

## Nomenclature

Symbol	Physical meaning
*S*	Common axial strain of the bonded layers
*E*_3_	Axial component of the electric field
*D*	Electric-displacement resultant per semiconductor cross-sectional area
*ϕ*	Electric potential
*H*_0_	Internal magnetic-field increment amplitude
*H*_b_	Internal magnetic bias defining the reference operating state
*m_H_*	Effective magnetic-field-to-electric-displacement coupling coefficient
*n*	Actual electron concentration
*p*	Actual hole concentration
*J^n^*	Axial electron current density
*J^p^*	Axial hole current density
$N_{D}^{+}$	Ionized donor concentration
$N_{A}^{-}$	Ionized acceptor concentration
*ε*_c_	Effective dielectric coefficient of the reduced composite model
*k*	Inverse carrier-screening length
*V*_T_	Thermal voltage
*A*	Dimensionless amplitude of the magnetic-field increment
Δ*V*_12_	Magnetically induced potential-difference increment between two fixed observation points
*S_V_*_,*H*_	Fixed-pair potential-difference response per unit magnetic-field increment
*ξ*	Half-spacing of a symmetric pair of fixed observation points
*D*_b_	Common electric-displacement increment prescribed at the two fiber ends
*η*_b_	Ratio of the end-displacement increment to the magnetic source
*f*_lat_	Outward lateral electric-displacement flux per unit axial length, divided by the semiconductor cross-sectional area
*τ*_tr_	Carrier transport-relaxation time
*τ*_rec_	Carrier recombination time
